# Intrinsic 1${T}^{{\prime} }$ phase induced in atomically thin 2*H*-MoTe_2_ by a single terahertz pulse

**DOI:** 10.1038/s41467-023-41291-w

**Published:** 2023-09-22

**Authors:** Jiaojian Shi, Ya-Qing Bie, Alfred Zong, Shiang Fang, Wei Chen, Jinchi Han, Zhaolong Cao, Yong Zhang, Takashi Taniguchi, Kenji Watanabe, Xuewen Fu, Vladimir Bulović, Efthimios Kaxiras, Edoardo Baldini, Pablo Jarillo-Herrero, Keith A. Nelson

**Affiliations:** 1 Department of Chemistry, Massachusetts Institute of Technology, Cambridge, MA 02139 USA; 2 Department of Physics, Massachusetts Institute of Technology, Cambridge, MA 02139 USA; 3 State Key Lab of Optoelectronic Materials and Technologies, Guangdong Province Key Laboratory of Display Material and Technology, School of Electronics and Information Technology, Sun Yat-sen University, Guangzhou, 510275 People’s Republic of China; 4 Department of Chemistry, University of California, Berkeley, CA 94720 USA; 5 Department of Physics, Harvard University, Cambridge, MA 02138 USA; 6 Department of Physics and Astronomy, Center for Materials Theory, Rutgers University, Piscataway, NJ 08854 USA; 7 Department of Electrical Engineering and Computer Science, Massachusetts Institute of Technology, Cambridge, MA 02139 USA; 8 School of Integrated Circuits, Peking University, Beijing, 100871 People’s Republic of China; 9 Center for Materials Science & Engineering, Massachusetts Institute of Technology, Cambridge, MA 02139 USA; 10 International Center for Materials Nanoarchitectonics, National Institute for Materials Science, 1-1 Namiki, Tsukuba, 305-0044 Japan; 11 Research Center for Functional Materials, National Institute for Materials Science, 1-1 Namiki, Tsukuba, 305-0044 Japan; 12 Ultrafast Electron Microscopy Laboratory, The MOE Key Laboratory of Weak-Light Nonlinear Photonics, School of Physics, Nankai University, Tianjin, 300071 People’s Republic of China; 13 Department of Physics, Center for Complex Quantum System, The University of Texas at Austin, Austin, TX 78712 USA; 14 Present Address: Department of Physics, Massachusetts Institute of Technology, Cambridge, MA 02139 USA

**Keywords:** Phase transitions and critical phenomena, Optical spectroscopy

## Abstract

The polymorphic transition from 2*H* to 1${T}^{{\prime} }$-MoTe_2_, which was thought to be induced by high-energy photon irradiation among many other means, has been intensely studied for its technological relevance in nanoscale transistors due to the remarkable improvement in electrical performance. However, it remains controversial whether a crystalline 1${T}^{{\prime} }$ phase is produced because optical signatures of this putative transition are found to be associated with the formation of tellurium clusters instead. Here we demonstrate the creation of an intrinsic 1${T}^{{\prime} }$ lattice after irradiating a mono- or few-layer 2*H*-MoTe_2_ with a single field-enhanced terahertz pulse. Unlike optical pulses, the low terahertz photon energy limits possible structural damages. We further develop a single-shot terahertz-pump-second-harmonic-probe technique and reveal a transition out of the 2*H*-phase within 10 ns after photoexcitation. Our results not only provide important insights to resolve the long-standing debate over the light-induced polymorphic transition in MoTe_2_ but also highlight the unique capability of strong-field terahertz pulses in manipulating quantum materials.

## Abstract (web summary)

A single field-enhanced terahertz pulse was used to create 1${T}^{{\prime} }$-MoTe_2_ out of few-layer *H*-MoTe_2_ crystals, providing insights into the ultrafast polymorphic transition dynamics and resolving a long-standing debate over the realization of this transition by high-energy photon irradiation.

## Introduction

**Fig. 1:**
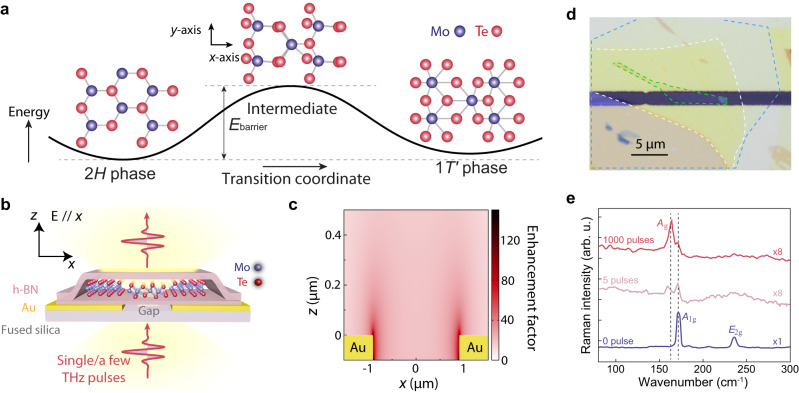
Polymorphic transition in MoTe_2_ induced by high-field THz pulses. **a** Schematic energy landscape and top view of lattice structures of 2*H*-MoTe_2_ (left), the intermediate state (middle), and 1${T}^{{\prime} }$-MoTe_2_ (right). *E*_barrier_ is the potential barrier between the 2*H* and 1${T}^{{\prime} }$-phase. **b** Cross-sectional schematic illustration of a 2*H*-MoTe_2_ crystal spanning an insulating gap between deposited gold strips, which serve as a THz field enhancement structure. The monolayer 2*H*-MoTe_2_ crystal is encapsulated between top and bottom h-BN. THz pulses are incident from the bottom side of the fused silica substrate. **c** Field strength calculation of the THz enhancement structure. Numerical simulation results showing THz field enhancement by a factor of 20–50 in significant regions in and near the gap between the gold strips. The enhancement factor is defined as the ratio between the actual and incident electric field. **d** Optical micrograph of a monolayer sample, including a 15-nm-thick top h-BN (white dashed line), a monolayer 2*H*-MoTe_2_ (green dashed line), and a 5-nm-thick bottom h-BN (blue dashed line), all spanning a 1.8-μm insulating gap (dark horizontal line) between the top and bottom gold strips. **e** Raman spectra of monolayer MoTe_2_ after successive THz pulse irradiation with free-space field amplitudes of 270 kV/cm. The *A*_g_ mode of the 1${T}^{{\prime} }$ phase sets in after 5 THz pulses and dominates upon further irradiation. By contrast, the *A*_1g_ and *E*_2g_ modes of the 2*H* phase are strongly reduced. The reduction of the overall Raman intensity in monolayer samples may be related to compromised crystallinity after the irradiation. The Raman measurements were conducted with a 1-μm-diameter laser spot and were therefore averaged over regions that had been subjected to differently enhanced THz field strengths. The two dashed lines are aligned with the *A*_g_ and *A*_1g_ mode of the 1${T}^{{\prime} }$ and 2*H*-phase, respectively.

Tailored, ultrashort pulses of light can be used to manipulate metastable states^1^ in functional materials, such as triggering insulator-to-metal transitions^2^, unveiling hidden states^3^, or even inducing long-lasting superconducting-like behavior way above the equilibrium critical temperature^4^. These photoinduced states have been intensely studied not only because they yield profound insights into fundamental light-matter interactions in correlated systems but also because they bring new opportunities to the field of laser fabrication and micro-machining, going beyond traditional laser cutting^5^, burning^6^, or stereolithography technologies^7^. From this application perspective, the metastable 1${T}^{{\prime} }$ phase of MoTe_2_ induced in a semiconducting 2*H* polymorph is considered an important candidate for tackling the issue of high contact resistance for two-dimensional electrical device^8^. In this polymorphic transition, the hexagonal unit cell of 2*H*-MoTe_2_ in its ground state is transformed into the metastable 1${T}^{{\prime} }$ phase after going through an intermediate structure (Fig. 1a), a process that was believed to be readily instigated by optical irradiation^9–13^. Compared to other methods^14,15^ such as ionic gating^16,17^ and strain application^18^, the focused laser spot down to the sub-micrometer regime makes an optically driven transition especially appealing for precision fabrication of small devices.

Despite the high technological impact of this polymorphic transition in MoTe_2_, recent studies have cast doubt on the existence of the purported 1${T}^{{\prime} }$ structure upon optical illumination. In particular, the main evidence for the 1${T}^{{\prime} }$ polymorph was the *A*_g_ phonon peaks around 120 cm^−1^ and 140 cm^−1^ in Raman spectroscopy^19,20^ (Supplementary Figs. 1 and 2), which have been shown to originate from nano- or micro-sized Te clusters. Given the high energy barrier *E*_barrier_ of more than 0.8 eV per MoTe_2_ formula unit^21^ as well as the large Te displacement of more than 1 Å required for the transition^22^ (Fig. 1a), it remains unclear whether light can initiate the polymorphic change after all.

In this work, instead of using visible light, we report the successful transition from 2*H* to 1${T}^{{\prime} }$-MoTe_2_ using single-cycle, field-enhanced terahertz (THz) pulses. The transition can be induced with as few as a single THz pulse on atomically thin crystals of 2*H*-MoTe_2_. Using Raman spectroscopy, we show that the induced phase reflects an intrinsic 1${T}^{{\prime} }$ structure^16,17,23^, which is free from any spurious features associated with Te clusters. The validity of the metastable 1${T}^{{\prime} }$ phase is further confirmed by selected area electron diffraction and energy-dispersive X-ray spectroscopy, which demonstrate a macroscopic structural transition without stoichiometric changes in the sample. Using single-shot, time-resolved nonlinear optical spectroscopy, we also unveil the different stages of this irreversible phase transition. These findings provide a route of structural manipulation using high-field THz pulses, which crucially avoid large-scale defect generation that often accompanies intense optical excitation^9^.

## Results

### Polymorphic transition in monolayer MoTe_2_

In our experiments, we positioned a flake of 2*H*-MoTe_2_ encapsulated by hexagonal-boron nitride (h-BN) on top of two parallel gold strips separated by a gap (Fig. 1b). The h-BN layers were used to isolate monolayers and bilayer MoTe_2_ samples from field-induced emission in the gold layers^24^ and to prevent their direct exposure to air. Figure 1d shows the optical image of a monolayer sample prepared using a dry transfer method^25^. Upon illumination of this structure with a free-space THz pulse, the field strength can be enhanced by more than 100 times at certain hot spots and about 20 times at the gap center, as shown in Fig. 1c. Since the peak electric field of the THz pulse in free space reached ~ 300 kV/cm at the focus, the electrical field amplitude can be more than 5 MV/cm in large parts of the sample^26^ (see Fig. 1c, Supplementary Fig. 3, “Methods” section, and Supplementary Note 1 for detailed information on THz generation). After irradiating this structure with a free-space THz field of 270 kV/cm, we probed lattice changes via spontaneous Raman scattering. Figure 1e shows the Raman spectra. Before the THz illumination, the spectrum consists of the *A*_1g_ phonon at 171.5 cm^−1^ and the *E*_2g_ phonon at 236 cm^−1^, in agreement with previous studies of the 2*H* phase^22^. After excitation with five THz pulses, both modes of the 2*H* phase disappear and a peak at 163.3 cm^−1^ emerges (see Supplementary Fig. 4 for additional peak width analysis). This feature signifies a change of the crystalline symmetry, and it is considered a specific fingerprint of the 1${T}^{{\prime} }$ phase of MoTe_2_^16,22^. The new phonon peak becomes more prominent after further exposure to THz pulses (Fig. 1e), indicating the growth of a larger 1${T}^{{\prime} }$ sample area. Importantly, the spectrum does not show any localized modes due to damage-related Te clusters^19^ even though the resultant $1{T}^{{\prime} }$ phase is not homogeneously produced in the flake suspended between the gap of the gold strips (Supplementary Figs. 5 and 6). These observations were reproduced in multiple samples, suggesting that the action of a strong THz field on our monolayer MoTe_2_ is to induce a phase transition from the 2*H* to the 1${T}^{{\prime} }$ polymorph.

**Fig. 2:**
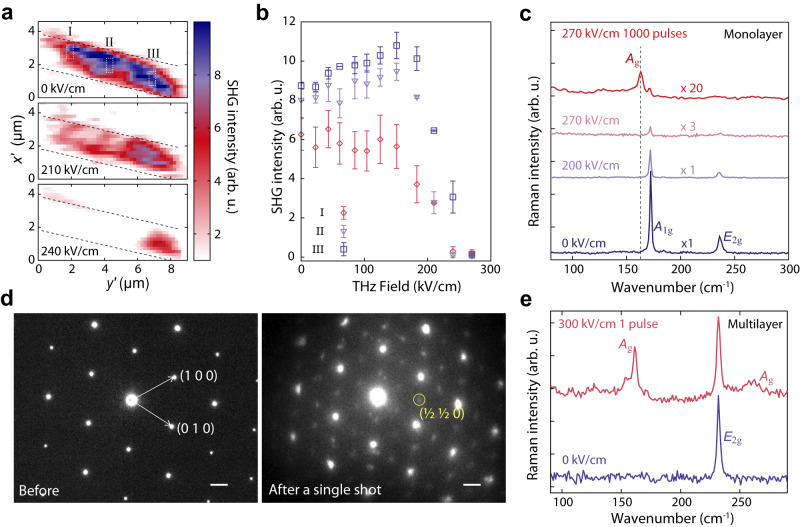
THz field dependence of the phase transition in monolayer and multilayer MoTe_2_. **a** SHG images of monolayer MoTe_2_ before THz irradiation (0 kV/cm), after irradiation with one THz pulse at 210 kV/cm free-space field amplitude, and after irradiation with a second THz pulse at 240 kV/cm. The dashed lines indicate the edges of gold strips in the field enhancement structure. Different areas of monolayer MoTe_2_ in the gap are outlined by the white dashed boxes and labeled by I, II, and III. The primes in ${x}^{{\prime} }$ and ${y}^{{\prime} }$ of the axes are added to differentiate the *x*–*y* coordinates in Fig. 1 due to the rotated field of view in SHG microscopy. **b** SHG from different areas (I–III) in **a** measured after irradiation of the sample by single THz pulses with successively increasing field strength. The vertical error bars measure the deviation of the averaged SHG intensity in the regions of interest from that of nearby pixels in the image. **c** Raman spectra of monolayer MoTe_2_ samples prior to THz irradiation (blue curve), after THz irradiation with a single pulse at 200 kV/cm (violet curve), another single pulse at 270 kV/cm (pink curve), and 1000 pulses at 270 kV/cm (red curve). The monolayer MoTe_2_ sample shows the *A*_g_ mode at 163.5 cm^−1^ after irradiation with 1000 pulses at 270 kV/cm. **d** Electron diffraction pattern of a multilayer ( ~ 10 layers) MoTe_2_ before and after a single THz pulse irradiation at 300 kV/cm, showing the emergence of superstructure peaks (yellow circle) that are characteristic of the $1{T}^{{\prime} }$ phase. The scale bars represent 0.1 Å^−1^. **e** Raman spectra of the multilayer MoTe_2_ before and after a single THz pulse irradiation at 300 kV/cm, showing the emergence of new Raman peaks that are characteristic of the induced 1${T}^{{\prime} }$ phase.

To further investigate this structural transformation, we conducted experiments on a series of monolayer 2*H*-MoTe_2_ samples. Since the induced 1${T}^{{\prime} }$ phase is long-lived, each measurement required a fresh specimen that was carefully positioned over the gap between the gold strips, as indicated in Fig. 1b. First, we studied how the 2*H* phase responded to a sequence of THz pulses with a gradually increasing field strength. To probe the phase transition, we tracked the changes in crystalline symmetry via optical second harmonic generation (SHG), a sensitive probe of inversion symmetry breaking that can distinguish between the non-centrosymmetric 2*H* phase and the centrosymmetric 1${T}^{{\prime} }$ phase in samples with an odd number of layers^27,28^ (see “Methods” section). Figure 2a shows three representative real-space images of the SHG intensity before and after THz-field exposures with free-space amplitudes of 210 kV/cm and 240 kV/cm; additional SHG intensity images are shown in Supplementary Fig. 7. A plot of the SHG intensities from three sample locations within the field-enhancement gap (labeled I, II, III in Fig. 2a) is presented in Fig. 2b. We observe that the SHG signal drops when the field strength exceeds 183 kV/cm, disappearing completely above 270 kV/cm. As the field enhancement factor generated by the gold strips is around 20, the threshold needed to quench the SHG intensity is estimated to be approximately 4.0 MV/cm. We complemented these measurements by recording Raman spectra as a function of the THz field strength. Figure 2c shows the spectra acquired after irradiation at 200 kV/cm and 270 kV/cm. Consistent with the picture supported by the SHG results in Fig. 2b, a single-pulse irradiation first leads to a drop in the intensity of the 2*H* phonons with a free-space field strength of 200 kV/cm, and stronger THz pulses at 270 kV/cm subsequently cause a further suppression, leading to a nearly complete extinction of the peak after 1000 pulses. Simultaneously, the Raman mode of the 1${T}^{{\prime} }$ phase grows, eventually dominating the spectrum.

### Polymorphic transition in multilayer MoTe_2_

The THz-induced transition is not restricted to monolayer samples, and we observed similar phenomenology in bilayer and multilayer (~10 layers) 2*H*-MoTe_2_ (see Supplementary Figs. 6c, d and 8a, b). Figure 2d, e show the results obtained on a multilayer 2*H*-MoTe_2_, presenting the [001] zone-axis electron diffraction pattern and Raman spectra before and after irradiation with a single THz pulse at 300 kV/cm. We find that the diffraction pattern of the unexcited crystal exhibits the six-fold symmetry expected for the 2*H* phase. Consistent with this observation, the Raman spectrum only displays an *E*_2g_ phonon mode of bulk 2*H*-MoTe_2_^29^. After THz irradiation, new peaks emerge in the diffraction pattern taken along the [001] zone axis of the new unit cell (Fig. 2d), revealing a cell-doubling superstructure^30–33^. On the other hand, our energy-dispersive X-ray spectroscopy mapping of the irradiated sample shows that the stoichiometric ratio of ~1: 2 is maintained and Te clusters are absent (Supplementary Fig. 9). The change in the electron diffraction pattern is accompanied by the appearance of *A*_g_ phonons (164 cm^−1^ and 270 cm^−1^) in the Raman spectrum (Fig. 2e)^16,19^. Both observables are characteristic signatures of the 1${T}^{{\prime} }$ lattice, offering additional validation for the creation of an intrinsic 1${T}^{{\prime} }$ phase (see Supplementary Note 2 and 3, and Supplementary Fig. 10).

### Temporal evolution of the polymorphic transition

**Fig. 3:**
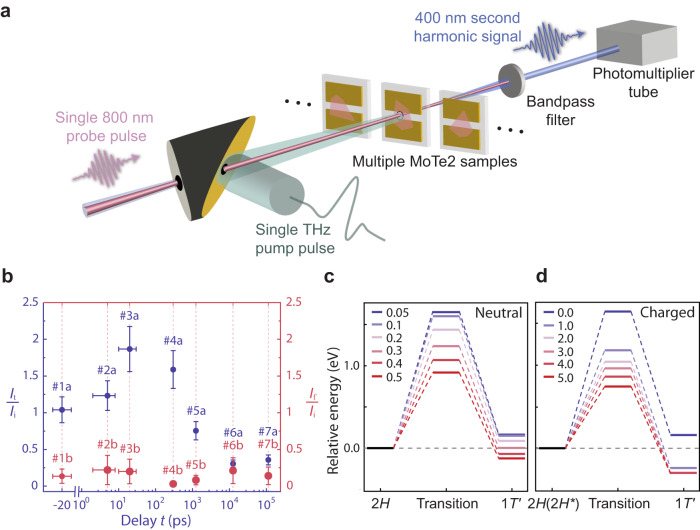
Dynamics and driving mechanism of the THz-field-induced phase transition. **a** Schematic illustration of the setup for single-shot SHG probe with a THz excitation pulse. Single-shot measurements were conducted by using a fresh sample in each shot. **b** SHG intensities measured from trilayer MoTe_2_ samples at different delay times *t* from several picoseconds to hundreds of nanoseconds (blue dots) as well as around 1 minute (red dots). The data points on the same dashed line show the SHG intensity measured from the same flake shortly after (*I*_t_) and 1 min after the THz excitation pulse (*I*_f_), normalized by the SHG signal intensity prior to THz excitation (*I*_i_). Due to the destructive nature of single-shot SHG, *I*_i_, *I*_t_, and *I*_f_ were measured at three different spots on the same sample. For this reason, we use #1a, #1b, etc. to emphasize that these data points were taken at different spots (a, b) of the same flake (#1). The error analysis is provided in Supplementary Note 4. **c** The energy potential as a function of transition coordinate with nonequilibrium carrier distribution (charge neutral). Nonequilibrium distribution of carriers is qualitatively described by the Fermi-smearing method. The free-energy barrier decreases from 1.64 eV to 0.91 eV as the Fermi-smearing width increases from 0.05 eV to 0.5 eV. The figure legend has a unit of eV. **d** The energy potential as a function of transition coordinate upon charge dopings. As the added charge density increases to 1.0 *e*/MoTe_2_, which corresponds to 9 × 10^14^ cm^−2^, the activation energy decreases from 1.66 eV to 1.19 eV. The figure legend has a unit of *e*/MoTe_2_. The atomic structures of transition states are shown in Supplementary Figs. 18 and 19.

To gain microscopic insights into the THz-driven transition, we also examined the photo-induced dynamics to trace out the temporal evolution of the crystalline lattice. This investigation cannot be accomplished by conventional time-resolved spectroscopy methods because their stroboscopic approach precludes the study of irreversible processes. We therefore developed a specialized apparatus for THz pump-SHG probe single-shot spectroscopy, which is well suited for probing the formation dynamics of the long-lived metastable 1${T}^{{\prime} }$ state. A schematic illustration of our experiment is presented in Fig. 3a; details of the setup are described in Methods. In our measurements, we irradiated trilayer samples with a single THz pulse, whose field strength was adjusted to exceed the phase transition threshold. We subsequently recorded the SHG signal at different pump-probe delays. Since the THz field was above the threshold for generating the 1${T}^{{\prime} }$ phase, the experiment was challenging because each THz shot with its predetermined delay time needed to be conducted on a fresh piece of sample. An added difficulty is the extremely weak single-shot SHG signal from a few-layer sample; using a photomultiplier tube, we obtained measurable second harmonic intensity under an incident 800-nm pulse fluence of ~ 20 mJ/cm^2^, which is beyond the optical damage threshold. Here, the collection of single-shot SHG signals relies on the concept of “probe before destruction”^34,35^, which is well applicable to MoTe_2_ (see Supplementary Note 4). The small beam spot for SHG (1 μm) relative to the sample size (≥10 μm) and good spatial uniformity of the trilayer crystals (Supplementary Fig. 11) allow a comparison among three locally destructive single-shot SHG measurements at different sample locations: one prior to THz excitation (*I*_i_), the second at the selected delay time following THz excitation (*I*_t_), and the third at a long delay time at ~1 min (*I*_f_).

Figure 3b shows the time evolution of the SHG signal relative to the initial signal following THz excitation (*I*_t_/*I*_i_, blue dots). The response first increases, reaching about twice the initial value at 20 ps. It then remains higher than *I*_i_ for several hundred picoseconds, before dropping to ~0.2*I*_i_ at 12.5 ns. Finally, at long pump-probe delays, the SHG signal disappears completely, indicating the stabilization of a metastable phase. The metastability is further confirmed by the value of the final signals after 1 min of photoexcitation, *I*_f_/*I*_i_, shown as the red dots in Fig. 3b. We were able to reproduce these complex dynamics on different samples, with the initial enhancement of the SHG occurring even below the phase transition threshold (Supplementary Fig. 12). From our single-shot spectroscopy data, we identify a characteristic timescale on the order of 10 ns that is needed to completely switch MoTe_2_ out of the 2*H* polymorph. We attribute the increase in SHG signal observed at shorter times to a possible nucleation of a transient structure with a lower crystalline symmetry than 2*H*. One such possibility is the distorted trigonal prismatic 2H^*^ phase^12,14^ (Supplementary Fig. 18), which is metastable along the transition pathway and can further prolong the transition timescale. This scenario at short delay times requires additional characterizations by single-shot time-resolved techniques beyond SHG, such as diffraction or visible-IR reflectivity to probe transient lattice and electronic responses, which are beyond the scope of our present study.

## Discussion

The set of experimental data from multiple techniques allows us to theoretically explore different effects that can account for the 2*H*-to-1${T}^{{\prime} }$ transition. Based on the nanosecond phase transition timescale, a natural hypothesis involves a THz field-driven carrier excitation mechanism. Microscopically, an intense electric field at THz frequency can generate high carrier densities through mechanisms such as Poole-Frenkel ionization, which liberate carriers and accelerate them to multi-eV energies (see Supplementary Note 5). These processes lead to impact ionization, liberating more carriers and evolving as a cascade event^36^. Such electron-hole generation can lead to either (i) a neutral carrier redistribution when excited carriers remain quasi-free, or (ii) charge doping when carriers localize at the substrate/h-BN interfacial states or spatially separate under the drive of intense THz electric fields especially in the presence of trap states^37^ (see Supplementary Note 5). To account for both scenarios, we numerically investigated the effects of neutral as well as charged carrier excitation on the energy landscape of MoTe_2_.

The results of our calculations are shown in Fig. 3c,d (see Methods and Supplementary Note 6 for details of the first-principles calculations). In equilibrium, the metastable 1${T}^{{\prime} }$ phase lies about 0.1 eV higher in energy than the 2*H* phase (blue horizontal lines); here, energy values are quoted per two formula units of MoTe_2_ due to unit cell doubling in this transition. The 2*H*-to-1${T}^{{\prime} }$ activation barrier and the reverse activation barrier at charge neutrality in equilibrium is 1.66 eV and 1.56 eV, respectively, thus protecting the 2*H* and 1${T}^{{\prime} }$ phases against thermal fluctuations. Upon excitation of a neutral electron-hole redistribution (Fig. 3c) or charge doping (Fig. 3d), two effects occur. First, the activation barrier lowers substantially (violet-to-red horizontal lines) and second, the 1${T}^{{\prime} }$ energy decreases, both effects favoring the occurrence of the 2*H*-to-1${T}^{{\prime} }$ transition.

Our joint experimental and theoretical studies establish intense THz fields as a viable route to steer the polymorphic transition in atomically thin MoTe_2_. Crucially, this transition cannot be induced by photons at higher energies^9–11,38^, as evidenced by our experiments performed with mid-infrared and near-infrared pulses centered around 225 meV and 1.55 eV, respectively (Supplementary Figs. 1 and 2). The key difference between mid- or near-infrared and THz pulses is that the former usually leads to mobile carriers at very high-energy conduction bands, and these high-energy mobile carriers will result in significant lattice heating — hence structural damage including Te cluster formation — through intraband relaxation. On the other hand, strong THz fields can create mobile carriers at the conduction band edge without significant lattice heating effect (see Supplementary Note 5). We also noticed that another mechanism involving THz-excited phonons was proposed to drive the 2*H*-1${T}^{{\prime} }$ phase transition in MoTe_2_^39^, which could also contribute to our observation uniquely driven by THz pulses.

The large structural distortion also leads to a significant electronic structure reconstruction involving band inversion around the Γ point, driving a topologically trivial 2*H* phase of few-layer MoTe_2_ into a quantum spin Hall insulator state in the 1${T}^{{\prime} }$ phase^40^ (see Supplementary Note 7). The capability demonstrated in this work hence opens the tantalizing prospect in the search for phases of matter with non-trivial band topology in addition to highlighting the unique capability of high-field THz pulses in shaping material properties in a complex, multi-phase energy landscape.

## Methods

### Fabrication of THz field-enhancement structure on fused silica substrate

The fabrication of the metal microslit array was based on a standard photolithography and lift-off process. Image reversal photoresist AZ5214 was spin-coated on a fused silica substrate at 3000 rpm for 30 s, soft baked at 110 °C for 50 s on a hotplate, UV exposed by a maskless aligner MLA 150 with a dose of 24 mJ/cm^2^, and post-exposure baked at 120 °C for 2 min followed by flood exposure and development in AZ422. A thin film of 2-nm Cr was deposited onto the substrate as an adhesion layer by thermal evaporation followed by a 98-nm-thick Au thin film. The sample was soaked in acetone and PG remover for lift-off to complete fabrication of the field enhancement structure.

### Layered MoTe_2_ integration with the field enhancement structure

The monolayer, few-layer MoTe_2_, and layered h-BN were exfoliated on SiO_2_/Si substrate from bulk MoTe_2_ (HQ graphene) or h-BN crystals. Monolayer and bilayer MoTe_2_ were identified by optical contrast and Raman spectroscopy. The layered materials were picked up by a transfer slide composed of a stack of glass, a polydimethylsiloxane (PDMS) film and a polycarbonate (PC) film^41^. The resulting stacks of top h-BN layer, MoTe_2_ monolayer or bilayer, and bottom h-BN layer were then placed on top of the field enhancement structure with the help of a transfer setup under an optical microscope^24^. For trilayer or thicker MoTe_2_ samples used in the SHG and electron microscopy measurements, h-BN encapsulation was not used.

### High-field THz pulse generation

High-field THz pulses were generated in a Mg:LiNbO_3_ crystal by tilting the optical pulse front of an 800-nm pump pulse to achieve phase matching^42^. By using a three-parabolic-mirror THz imaging system, the image of the THz beam spot on the sample was focused to its diffraction limit of around 500 μm in diameter. The incident THz pulse temporal profile was measured in the time domain using electro-optic sampling with a 100-μm-thick 110-oriented GaP crystal. When pumping with the 4 W output from an amplified Ti:sapphire laser system (repetition rate 1 kHz, central wavelength 800 nm, pulse duration 100 fs), the peak electric field of the THz pulses reached ~ 300 kV/cm at the focus, with a spectrum centered at around 0.5 THz (see Supplementary Figs. 13 and 14). The repetition rate of the laser output was down-counted from 1 kHz to 125 Hz by three successive phase-locked choppers, providing sufficient temporal separation between pulses for a mechanical shutter to isolate single THz pulses.

### Spontaneous Raman scattering measurements

Spontaneous Raman scattering was performed on samples before and after THz irradiation using a commercial Raman spectrometer (Horiba LabRAM) with a HeNe laser (*λ* = 632.8 nm). The laser beam was focused on the samples by a 100×  objective into a spot with a diameter of approximately 1 μm.

### Transmission electron diffraction microscopy

To prepare the samples for transmission electron microscopy (TEM) characterization before and after THz excitation, we designed a special TEM grid with a THz field enhancement structure. The enhancement pattern made of a 50-nm-thick gold film is deposited on a silicon nitride TEM window. The enhancement structure is a 300-μm-long, 2-μm-wide air gap within the gold film, as shown in Supplementary Fig. 10. We transferred a multilayer MoTe_2_ flake on top of the gap using a PDMS dry-transfer method^43^ and measured the diffraction pattern before and after the single THz pulse excitation. The transmission electron diffraction measurement was performed using a transmission electron microscope (FEI Tecnai Multipurpose Digital TEM) operated at 60 keV at room temperature.

### SHG mapping

As shown in Supplementary Fig. 14, the SHG fundamental pulses were provided by a mode-locked Ti:sapphire oscillator centered at 800 nm. The laser pulse duration was around 35 fs at an 80 MHz repetition rate. The pulse was linearly polarized by an achromatic polarizer (400–800 nm) and the polarization at the sample was controlled by an achromatic half-wave plate in a motorized rotational stage. SHG signals from the sample were collected by the same objective for focusing the fundamental light, and they transmitted through the same half-wave plate and polarizer, which ensured that the SHG components detected were parallel to the polarization of the fundamental field. A photomultiplier tube (PMT, Hamamatsu Photonics H10721) was used to analyze the SHG signal. In the fast mapping mode, the laser beam on the sample was scanned using two-axis Galvo mirrors (Thorlabs, GVS412) to acquire in situ SHG images. We also use the SHG mapping method to look for trilayer samples which have uniform response as shown in Supplementary Fig. 11 for the single-shot THz pump-SHG probe measurements.

### Single-shot THz pump-SHG probe microscopy

The THz pump arm was combined with SHG pulses from a second, temporally synchronized 12 W amplified Ti:sapphire laser (repetition rate 1 kHz, central wavelength 800 nm, pulse duration 35 fs). SHG light was focused to a near-diffraction-limited size (1 μm) at the sample with a 50× objective. The SHG light was collected by the same objective and detected by a PMT with a confocal microscope to selectively probe the area at the focus with single-optical-pulse irradiation. The power level of the SHG excitation pulse was well above the damage threshold of MoTe_2_, but the ultrafast nature of the pulse enabled us to obtain a reliable SHG signal before the sample was damaged. The temporal overlap of the counter-propagating THz field and SHG optical pulse was determined by THz field-induced second harmonic signal from a 30-μm-thick LiNbO_3_ slab. All of the optical measurements were conducted on a single-shot basis under ambient conditions. More details are given in Supplementary Note 4 and Supplementary Figs. 11d, 15, and 16.

### First-principles calculations

We performed density functional theory (DFT) calculations using the VASP code^44,45^ within the general gradient approximation (GGA) — Perdew-Burke-Ernzerhof (PBE) exchange-correlation functional^46^. The kinetic energy cutoff was 350 eV for the plane-wave basis sets. The lattice constants of 2*H* and 1${T}^{{\prime} }$ phases in the neutral state were obtained via structural optimization with a convergence threshold of force on each atom of 0.01 eV/Å, with a vacuum region more than 36-Å-thick to decouple the neighboring slabs. A Γ-centered 11 × 17 × 1*k*-point mesh was used to sample the Brillouin zone. The climbing image nudged elastic band (CI-NEB) method^47^ with 5 to 7 intermediate images was used to determine the activation barrier of the phase transition. Besides converging the electronic ground states with charge neutrality, we also considered higher temperature smearing effects and charge doping. More information can be found in Supplementary Note 6 and Supplementary Figs. 17–20.

In these barrier calculations, the in-plane lattice constants were all constrained to the value of the neutral state 2*H* phase, even for the charged state conditions in which the lattice tends to expand. We note that the treatment of constant area was mainly due to the difficulty of convergence when the lattice vectors were allowed to relax during CI-NEB calculations of charged slabs. It simulates the situation where the lattice of monolayer MoTe_2_ is strongly constrained by its interaction with substrates.

Based on the converged electronic ground state with spin-orbit coupling in a given crystal structure, we further constructed the Wannier electronic model using the Wannier90 code^48,49^ by projecting the Mo d-orbitals and Te p-orbitals near the Fermi level. Besides capturing the electronic band structure, the Wannier models also allowed us to investigate the topological properties by evaluating the Z_2_ topological index from the non-Abelian Berry connections along the Wilson loop^50^ and the Fu-Kane parity formulation^51^ (when inversion symmetry is present). More information can be found in Supplementary Figs. 21–23.

### Supplementary information

Supplementary Information

## Data Availability

Relevant data supporting the key findings of this study are available within the article and the Supplementary Information file. All raw data generated during the current study are available from the corresponding authors upon request.

## References

[1] Sie EJ, Nyby CM, Pemmaraju CD, Park SJ, Shen X (2019). An ultrafast symmetry switch in a Weyl semimetal. Nature.

[2] Liu M, Hwang HY, Tao H, Strikwerda AC, Fan K (2012). Terahertz-field-induced insulator-to-metal transition in vanadium dioxide metamaterial. Nature.

[3] Stojchevska L, Vaskivskyi I, Mertelj T, Kusar P, Svetin D (2014). Ultrafast switching to a stable hidden quantum state in an electronic crystal. Science.

[4] Budden M, Gebert T, Buzzi M, Jotzu G, Wang E (2021). Evidence for metastable photo-induced superconductivity in K_3_C_60_. Nat. Phys..

[5] Niziev VG, Nesterov AV (1999). Influence of beam polarization on laser cutting efficiency. J. Phys. D Appl. Phys..

[6] Buchalla W, Attin T (2007). External bleaching therapy with activation by heat, light or laser—a systematic review. Dent. Mater..

[7] Popov VK, Evseev AV, Ivanov AL, Roginski VV, Volozhin AI (2004). Laser stereolithography and supercritical fluid processing for custom-designed implant fabrication. J. Mater. Sci.: Mater. Med..

[8] Zhang X, Jin Z, Wang L, Hachtel JA, Villarreal E (2019). Low contact barrier in 2*H*/1$${T}^{{\prime} }$$ MoTe_2_ in-plane heterostructure synthesized by chemical vapor deposition. ACS Appl. Mater. Interfaces.

[9] Cho S, Kim S, Kim JH, Zhao J, Seok J (2015). Phase patterning for ohmic homojunction contact in MoTe_2_. Science.

[10] Song Y, Tian R, Yang J, Yin R, Zhao J (2018). Second harmonic generation in atomically thin MoTe_2_. Adv. Opt. Mater..

[11] Tan Y, Luo F, Zhu M, Xu X, Ye Y (2018). Controllable 2H-to-1T$${}^{{\prime} }$$ phase transition in few-layer MoTe_2_. Nanoscale.

[12] Kolobov AV, Fons P, Tominaga J (2016). Electronic excitation-induced semiconductor-to-metal transition in monolayer MoTe_2_. Phys. Rev. B.

[13] Peng B, Zhang H, Chen W, Hou B, Qiu Z-J (2020). Sub-picosecond photo-induced displacive phase transition in two-dimensional MoTe_2_. npj 2D Mater. Appl..

[14] Zhang F, Zhang H, Krylyuk S, Milligan CA, Zhu Y (2019). Electric-field induced structural transition in vertical MoTe_2_- and Mo_1−*x*_W_*x*_Te_2_-based resistive memories. Nat. Mater..

[15] Qi Y, Naumov PG, Ali MN, Rajamathi CR, Schnelle W (2016). Superconductivity in Weyl semimetal candidate MoTe_2_. Nat. Commun..

[16] Wang Y, Xiao J, Zhu H, Li Y, Alsaid Y (2017). Structural phase transition in monolayer MoTe_2_ driven by electrostatic doping. Nature.

[17] Wang Y, Xiao J, Chung T-F, Nie Z, Yang S (2021). Direct electrical modulation of second-order optical susceptibility via phase transitions. Nat. Electron..

[18] Hou W, Azizimanesh A, Sewaket A, Peña T, Watson C (2019). Strain-based room-temperature non-volatile MoTe_2_ ferroelectric phase change transistor. Nat. Nanotechnol..

[19] Chen S-Y, Naylor CH, Goldstein T, Johnson ATC, Yan J (2017). Intrinsic phonon bands in high-quality monolayer T$${}^{{\prime} }$$ molybdenum ditelluride. ACS Nano.

[20] Manjón FJ, Gallego-Parra S, Rodriguez-Hernandez P, Munoz A, Drasar C (2021). Anomalous Raman modes in tellurides. J. Mater. Chem. C.

[21] Duerloo K-AN, Li Y, Reed EJ (2014). Structural phase transitions in two-dimensional Mo-and W-dichalcogenide monolayers. Nat. Commun..

[22] Keum DH, Cho S, Kim JH, Choe D-H, Sung H-J (2015). Bandgap opening in few-layered monoclinic MoTe_2_. Nat. Phys..

[23] Wang J, Luo X, Li S, Verzhbitskiy I, Zhao W (2017). Determination of crystal axes in semimetallic $${T}^{{\prime} }$$-MoTe_2_ by polarized Raman spectroscopy. Adv. Funct. Mater..

[24] Dean CR, Young AF, Meric I, Lee C, Wang L (2010). Boron nitride substrates for high-quality graphene electronics. Nat. Nanotechnol..

[25] Wang L, Meric I, Huang PY, Gao Q, Gao Y (2013). One-dimensional electrical contact to a two-dimensional material. Science.

[26] Pein BC, Chang W, Hwang HY, Scherer J, Coropceanu I (2017). Terahertz-driven luminescence and colossal Stark effect in CdSe-CdS colloidal quantum dots. Nano Lett..

[27] Yamamoto M, Wang ST, Ni M, Lin Y-F, Li S-L (2014). Strong enhancement of Raman scattering from a bulk-inactive vibrational mode in few-layer MoTe_2_. ACS Nano.

[28] Beams R, Cançado LG, Krylyuk S, Kalish I, Kalanyan B (2016). Characterization of few-layer 1$${T}^{{\prime} }$$ MoTe_2_ by polarization-resolved second harmonic generation and Raman scattering. ACS Nano.

[29] Ruppert C, Aslan OB, Heinz TF (2014). Optical properties and band gap of single- and few-layer MoTe_2_ crystals. Nano Lett..

[30] Wang L, Xu Z, Wang W, Bai X (2014). Atomic mechanism of dynamic electrochemical lithiation processes of MoS_2_ nanosheets. J. Am. Chem. Soc..

[31] Gao P, Wang L, Zhang Y, Huang Y, Liu K (2015). Atomic-scale probing of the dynamics of sodium transport and intercalation-induced phase transformations in MoS_2_. ACS Nano.

[32] Heising J, Kanatzidis MG (1999). Exfoliated and restacked MoS_2_ and WS_2_: Ionic or neutral species? Encapsulation and ordering of hard electropositive cations. J. Am. Chem. Soc..

[33] Meng L, Ma Y, Si K, Xu S, Wang J (2019). Recent advances of phase engineering in group VI transition metal dichalcogenides. Tungsten.

[34] Chapman HN, Fromme P, Barty A, White TA, Kirian RA (2011). Femtosecond X-ray protein nanocrystallography. Nature.

[35] Tenboer J, Basu S, Zatsepin N, Pande K, Milathianaki D (2014). Time-resolved serial crystallography captures high-resolution intermediates of photoactive yellow protein. Science.

[36] Fan K, Hwang HY, Liu M, Strikwerda AC, Sternbach A (2013). Nonlinear terahertz metamaterials via field-enhanced carrier dynamics in GaAs. Phys. Rev. Lett..

[37] Ju L, Velasco J, Huang E, Kahn S, Nosiglia C (2014). Photoinduced doping in heterostructures of graphene and boron nitride. Nat. Nanotechnol..

[38] Krishnamoorthy A, Lin M-F, Zhang X, Weninger C, Ma R (2019). Optical control of non-equilibrium phonon dynamics. Nano Lett..

[39] Zhou J, Xu H, Shi Y, Li J (2021). Terahertz driven reversible topological phase transition of monolayer transition metal dichalcogenides. Adv. Sci..

[40] Qian X, Liu J, Fu L, Li J (2014). Quantum spin Hall effect in two-dimensional transition metal dichalcogenides. Science.

[41] Zomer PJ, Guimarães MHD, Brant JC, Tombros N, van Wees BJ (2014). Fast pick up technique for high quality heterostructures of bilayer graphene and hexagonal boron nitride. Appl. Phys. Lett..

[42] Hebling J, Yeh K-L, Hoffmann MC, Bartal B, Nelson KA (2008). Generation of high-power terahertz pulses by tilted-pulse-front excitation and their application possibilities. J. Opt. Soc. Am. B.

[43] Bie Y-Q, Zong A, Wang X, Jarillo-Herrero P, Gedik N (2021). A versatile sample fabrication method for ultrafast electron diffraction. Ultramicroscopy.

[44] Kresse G, Furthmüller J (1996). Efficient iterative schemes for ab initio total-energy calculations using a plane-wave basis set. Phys. Rev. B.

[45] Kresse G, Furthmüller J (1996). Efficiency of ab-initio total energy calculations for metals and semiconductors using a plane-wave basis set. Comput. Mater. Sci..

[46] Perdew JP, Burke K, Ernzerhof M (1996). Generalized gradient approximation made simple. Phys. Rev. Lett..

[47] Henkelman G, Uberuaga BP, Jónsson H (2000). A climbing image nudged elastic band method for finding saddle points and minimum energy paths. J. Chem. Phys..

[48] Mostofi AA, Yates JR, Lee Y-S, Souza I, Vanderbilt D (2008). wannier90: a tool for obtaining maximally-localised Wannier functions. Comput. Phys. Commun..

[49] Mostofi AA, Yates JR, Pizzi G, Lee Y-S, Souza I (2014). An updated version of wannier90: a tool for obtaining maximally-localised Wannier functions. Comput. Phys. Commun..

[50] Yu R, Qi XL, Bernevig A, Fang Z, Dai X (2011). Equivalent expression of $${{\mathbb{Z}}}_{2}$$ topological invariant for band insulators using the non-Abelian Berry connection. Phys. Rev. B.

[51] Fu L, Kane CL (2007). Topological insulators with inversion symmetry. Phys. Rev. B.

