# Supplementary material for: Intrinsic 1T′ phase induced in atomically thin 2H-MoTe2 by a single terahertz pulse
Source: Nat Commun. 2023 Sep 22;14:5905. doi: 10.1038/s41467-023-41291-w (PMC10516973; doi:10.1038/s41467-023-41291-w)
Supplement: Supplementary file 1 — Supplementary Information [file 41467_2023_41291_MOESM1_ESM.pdf]

Supplementary Information for "Intrinsic $1T'$ phase induced in atomically thin $2H$-$MoTe_2$ by a single terahertz pulse"

Jiaojian Shi,[1,*] Ya-Qing Bie,[2,3,*,✉] Alfred Zong,[2,4,*] Shiang Fang,[5,6,14] Wei Chen,[5] Jinchi Han,[7,8] Zhaolong Cao,[3] Yong Zhang,[9] Takashi Taniguchi,[10] Kenji Watanabe,[11] Xuewen Fu,[12] Vladimir Bulović,[7] Efthimios Kaxiras,[5] Edoardo Baldini,[13] Pablo Jarillo-Herrero,[2,✉] Keith A. Nelson[1,✉]

[1]*Department of Chemistry, Massachusetts Institute of Technology, Cambridge, MA 02139, USA*

[2]*Department of Physics, Massachusetts Institute of Technology, Cambridge, MA 02139, USA*

[3]*State Key Lab of Optoelectronic Materials and Technologies, Guangdong Province Key Laboratory of Display Material and Technology, School of Electronics and Information Technology, Sun Yat-sen University, Guangzhou, 510275, People's Republic of China*

[4]*Department of Chemistry, University of California, Berkeley, CA 94720, USA*

[5]*Department of Physics, Harvard University, Cambridge, MA 02138, USA*

[6]*Department of Physics and Astronomy, Center for Materials Theory, Rutgers University, Piscataway, NJ 08854, USA*

[7]*Department of Electrical Engineering and Computer Science, Massachusetts Institute of Technology, Cambridge, MA 02139, USA*

[8]*School of Integrated Circuits, Peking University, Beijing, 100871, People's Republic of China*

[9]*Center for Materials Science & Engineering, Massachusetts Institute of Technology, Cambridge, MA 02139, USA*

[10]*International Center for Materials Nanoarchitectonics, National Institute for Materials Science, 1-1 Namiki, Tsukuba 305-0044, Japan*

[11]*Research Center for Functional Materials, National Institute for Materials Science, 1-1 Namiki, Tsukuba 305-0044, Japan*

[12]*Ultrafast Electron Microscopy Laboratory, The MOE Key Laboratory of Weak-Light Nonlinear Photonics, School of Physics, Nankai University, Tianjin, 300071, People's Republic of China*

[13]*Department of Physics, Center for Complex Quantum System, The University of Texas at Austin, Austin, TX 78712, USA*

[14]*Present address: Department of Physics, Massachusetts Institute of Technology, Cambridge, MA 02139, USA*

[*]These authors contributed equally to this work: Jiaojian Shi, Ya-Qing Bie, and Alfred Zong.

[✉]Correspondence to: Y.-Q.B. (bieyq@mail.sysu.edu.cn), P.J.-H. (pjarillo@mit.edu), K.A.N. (kanelson@mit.edu).

Supplementary Note 1. THz field enhancement structure simulation

The field of the free-space terahertz (THz) radiation can be enhanced with a metal microslit on a fused silica substrate as shown in Fig. 1c. Numerical simulations using commercial finite-difference time-domain (FDTD) software (FDTD solutions, Lumerical Inc.) were performed to calculate the enhancement factor of the gold slits. The dielectric function of gold in the THz frequency range was approximated by the Drude model,

$$\varepsilon = \varepsilon_\infty - \frac{\omega_p^2}{\omega^2 + i\omega\gamma}, \tag{S1}$$

where parameter values were taken from ref.[1]: $\varepsilon_\infty = 9.1$, $\omega_p = 1.38 \times 10^{16}$ rad/s, $\gamma = 1.075 \times 10^{14}$ rad/s; $n = 1.96$ was used for fused silica[2]. A THz light source was incident from the substrate side with an electric field along the x direction, as shown in Fig. 1b. Perfectly matched layers (PML) were used to absorb the scattered radiation in all directions. In the simulation, a power monitor was used to record the electric field along the gold surface. Numerical simulation results showing THz field enhancement by a factor of about 20 in significant regions in and near the gap between the gold strips. Supplementary Fig. 3 shows additional field-enhancement calculation with the presence of bulk $2H$-$MoTe_2$ with varying thicknesses up to 10-nm. Although the enhancement factor decreases for thicker samples, it remains high even with 10-nm $MoTe_2$, thereby indicating that the transition may still happen for thicker samples. The refractive index of the bulk $2H$-$MoTe_2$ at the THz frequency was taken from ref.[3]. Note that we did not consider the imaginary part of the dielectric function of $2H$-$MoTe_2$ that may lead to calculation deviations.

Supplementary Note 2. Transmission electron diffraction measurement

After a single THz excitation, new superstructure peaks emerge in the electron diffraction pattern, revealing a unit cell doubling. Similar superstructures in transmission electron diffraction have been observed in various chemical intercalation-induced phase transitions in MoS_2[4,5]. In these experiments, ions (Na^+ or Li^+) were intercalated between MoS_2 planes and they induced a phase transition from hexagonal ($2H$) to superstructured octahedral ($1T$) phase with ordered lattice distortions (i.e., $1T'$ phase[6]). The authors ascribed the 2×2 a-b plane superstructure reflections to the distorted $1T$ (i.e., $1T'$) phase[4,5,7]. In ref.[4],

the authors also measured the domain size of the $1T'$ phase to be $\sim$ 3–5 nm and found three different crystallographic orientations, with axes rotated by 120° (inherited from the symmetry of the $2H$ phase). Our electron diffraction pattern was taken along the [001] zone-axis. We estimated the in-plane domain size from the full width at half maximum of the diffraction spots, establishing a lower bound domain size of $\sim$ 10 nm. Due to inhomogeneity of the transition, the diffraction pattern of THz-irradiated $MoTe_2$ multilayers can also be understood by superimposing diffraction patterns from the $2H$ and $1T'$ phases[8] with three 120°-rotated crystallographic orientations originated from the $2H$ structure[4]. We also note that the label (1/2 1/2 0) is assigned to the peak circled in Fig. 2d as it appears between the (1 0 0) and (0 1 0) peaks and is indexed to the original $2H$-phase observed along the [0 0 1] zone axis.

Supplementary Note 3. Sample degradation and defect-induced phase transition

Unlike optical or infrared excitations, the THz-induced sample degradation does not serve as the main trigger for the phase transition. Spontaneous Raman scattering performed under our conditions is very sensitive to localized phonon modes around defect states, but our spectra are devoid of Te-cluster peaks[9] at 121 cm^{-1} and 142 cm^{-1}. From the electron diffraction measurements, we observed new superstructure peaks corresponding to the $1T'$ phase. This indicates that the sample is a crystalline phase with reasonably high quality.

In terms of the metastable phase transition mechanism, whether vacancies play a vital role as suggested in the optical process in ref.[10] remains an open question. We have performed MIR and NIR laser excitation and observed the same Raman spectra reported in refs.[11–14]. We believe all of these excitation methods induce vacancies as evident in the Raman spectrum, but none of them show the $2H$-to-$1T'$ phase transition in TEM diffraction measurements. Therefore, although theoretical calculations in ref.[10] propose vacancies are essential, our experiments indicate that vacancies cannot be the only trigger.

Supplementary Note 4. Single-shot THz pump-SHG probe microscopy

To obtain direct time-resolved observation of the metastable phase, we developed a setup for single-shot THz pump-SHG probe microscopy via sample replenishment in each shot.

Here we use trilayer $MoTe_2$ mainly because it is easier to prepare flakes with large enough area for the destructive pump-probe measurement which requires multiple shots to unveil the dynamics. The trilayer samples used here were not encapsulated with h-BN because the confined space between h-BN and $MoTe_2$ can explode after above-damage-threshold optical pulse excitation which may vaporize the materials inside. The methodology of "probe before destruction" has been demonstrated in various contexts including single-shot femtosecond X-ray nano-crystallography of radiation-sensitive biological systems[15–20]. In our case, the 800-nm pulse duration of 35 fs was faster than the onset of significant lattice motion in $MoTe_2$, which has been estimated to be between 150 fs and 1 ps[21,22]. The reliability of this method was confirmed by measurements of SHG as a function of the optical polarization angle relative to the crystallographic axes. The results of single-shot measurements at high fluence were consistent with those at low fluence and with the 6-fold rotational symmetry expected for a hexagonal phase 2D crystal (details are given in Supplementary Fig. 11d). The relatively large size and good uniformity of the trilayer crystals were sufficient to allow comparison for each flake among three locally destructive single-shot SHG measurements at different sample locations: one prior to THz excitation (initial signal intensity labeled I_{i}), a second at the selected delay time following the THz excitation pulse (I_{t}), and a third at a long time (approximately 1 minute, final intensity I_{f}) after THz excitation (see Supplementary Fig. 16). The delay times t were −20 ps, 5 ps, 20 ps, 300 ps, 1.24 ns, 12.5 ns and 112.5 ns for different samples.

We constructed an apparatus based on two synchronized chirped pulse amplifiers (CPAs) seeded by the same Ti:Sapphire oscillator (80 MHz, 800 nm), as shown in Supplementary Fig. 15. The repetition rate of the two amplifiers was 1 kHz. The central wavelength was 800 nm. One CPA output was 4 mJ energy per pulse with 100 fs pulse duration, while the other CPA output was 12 mJ with 35 fs pulse duration. The 4-mJ pulses from the 100-fs amplifier were used to generate high-field THz pulses in a $Mg{:}LiNbO_3$ crystal by the tilted pulse front technique. The THz pump arm was combined with the fundamental beam from the other synchronized 12-mJ (35 fs) Ti:sapphire CPA (used to produce SHG from the sample). The use of two synchronized CPAs as pump and probe arms enabled an arbitrary electronic timing adjustment between pump-probe delays. Fine tuning (< 12.5 ns) of the delay was realized by routing the beam via a translation stage. The SHG fundamental light was focused to an 1.5-μm spot size on the sample through a

50× objective, reaching a fluence of 20 mJ/cm^2. The SHG light was then collected by the same objective and detected by a photomultiplier tube (PMT) with a confocal microscope to selectively probe the area under single optical pulse irradiation. The power level of SHG excitation pulse was well above the damage threshold of 2D $MoTe_2$, but the ultrafast nature of the pulse enabled us to obtain an adequate SHG signal before the sample was damaged permanently by the probe pulse. The temporal overlap of the counter-propagating THz field and SHG optical pulse was determined by THz field-induced second harmonic signal of a thin $LiNbO_3$ slab (30 μm in thickness) that was placed in the sample location. All of the optical measurements were conducted on a single-shot basis at ambient conditions.

The two primary noise sources in our experimental measurements come from shot-to-shot SHG noise and sample variations. The shot-to-shot laser power noise was around 3–5%. In the second-order SHG process, the detected shot-to-shot SHG noise was around 6–10%. Because of the fluctuations in the electron multiplication process in the PMT, this noise was further increased to around 10–15%. The use of an objective could also cause additional noise from pointing fluctuations or sample vibrations near the focal plane. The choice of a long-working-distance (20 mm) 50× objective limited the noise from the objective to be less than 1–2%, which is negligible compared to the shot-to-shot SHG noise. Control over the signal fluctuations arising from sample variations was challenging due to the non-uniform nature[23] of mechanical exfoliation 2D $MoTe_2$. We checked SHG images and selected regions with the most uniform SHG distribution for all the single-shot dynamics measurements (Supplementary Fig. 11). In this way, SHG fluctuations from sample variations were controlled to be around 4% (estimated from SHG image in Supplementary Fig. 11b). The error bars in Fig. 3b take the above noise sources and SHG signal strengths into account.

Supplementary Note 5. Poole-Frenkel effect and impact ionization

In this section, we first present estimates of the extent of non-equilibrium excitation of electron and hole pairs (charge-neutral) with a THz electric field. Next, we analyze the possibility of the existence of charged states after THz pulse excitation due to a release of trapped charges at the h-BN/fused silica interface and an instantaneous spatial charge separation in the $MoTe_2$ layers.

With a THz pulse, excited electron-hole pairs can be induced through the Poole-Frenkel

effect and impact ionization. As shown in Supplementary Fig. 17a, the Poole-Frenkel (PF) effect describes carrier liberation via tunneling of bound carriers. It is facilitated by defect sites at which the binding energy is reduced. Since both electrons and holes can be excited in $MoTe_2$, the flake remains charge-neutral. This is different from the ionic gating case, where one type of charge is trapped at the surfaces by the gate voltage, leaving the flake charged with another type of carrier. Carrier liberation via the PF effect driven by metamaterial-enhanced THz fields (several MV/cm) has been observed in a correlated electron material, which initiated an insulator-to-metal transition[24]. Here we estimate the liberated carrier density in 2D $MoTe_2$ through the PF effect. Due to the ubiquitous existence of defects in 2D materials, with a typical density[25–27] of around 10^{13} cm^{-2}, the thermionic emission of trapped carriers gives rise to a carrier density of $n_0 \approx 10^9 - 10^{11}$ cm^{-2} at room temperature without an external electric field[28–30]. Since the electron density in the conduction band is small compared to the defect density, carrier liberation and conductivity enhancement due to an external electric field can be described by the following equation[31,32]:

$$\sigma = \sigma_0 e^{\left(\frac{e^3|E|}{\pi\epsilon\epsilon_0}\right)^{\frac{1}{2}}/k_B T} \tag{S2}$$

where σ is the conductivity in an external electric field of strength $|E|$, σ_0 is the field-free conductivity, ϵ_0 is the vacuum permittivity, ϵ is the relative DC dielectric constant, k_B is the Boltzmann constant, and T is the temperature. At our highest free-space THz field strength of 270 kV/cm, the in-gap field strength is greater than 5 MV/cm at the center of the gap. The field can reach 27 MV/cm at the gap edge[33] according to the simulation, but the highly confined spot has negligible contribution to the effective carrier dynamics. Electric fields at a level of several MV/cm field strength are well within the high-field regime for semiconductors with sub-eV to several-eV band gaps, where the carrier drift velocities v_s strongly deviate from linear dependence on the applied field and reach saturation velocity[34].

Assuming the carrier drift velocity reaches saturation velocity at our in-gap THz electric field, the field-liberated free electron density n is given by

$$n = \frac{\sigma}{e\mu_s} = \frac{\sigma_0}{e\mu_s} e^{\frac{\left(\frac{e^3|E|}{\pi\epsilon\epsilon_0}\right)^{\frac{1}{2}}}{k_B T}} = \frac{\mu_0}{\mu_s} n_0 e^{\frac{\left(\frac{e^3|E|}{\pi\epsilon\epsilon_0}\right)^{\frac{1}{2}}}{k_B T}} \tag{S3}$$

where $n_0 \approx 10^9$–10^{11} cm^{-2} is the field-free carrier density due to thermionic emission of carriers in shallow traps typical of 2D materials, μ_0 is the carrier mobility before THz excitation, and $\mu_s < \mu_0$ is the mobility in the field-driven sample. The relative DC dielectric constant[3] in 2D $MoTe_2$ is around 20. Those values give us a lower bound of the field-liberated carrier density with $\left(\frac{e^3|E|}{\pi\epsilon\epsilon_0}\right)^{\frac{1}{2}}/k_BT > 14$, which corresponds to $n > \frac{\mu_0}{\mu_s} \times n_0 \times 10^6 > n_0 \times 10^6 > 10^{15}$ cm^{-2}. This estimate does not account for any limit on the total number of electrons in the states from which thermionic emission occurs. Since the PF effect in our case is directly related to the defect densities which are typically on the order of 10^{13} cm^{-2} (mostly point defects) in mechanically exfoliated 2D materials[26], our estimate indicates that the carriers in shallow traps are completely liberated via the PF effect. Assuming most defects support no more than one trapped electron, the lower bound of the carrier density is given by the defect density of 10^{13} cm^{-2}.

It is also well established that enhanced THz fields can cause extensive impact ionization (IMI). For example, the carrier density in GaAs was increased by roughly a billion-fold within a metamaterial insulating gap upon THz irradiation[35]. As illustrated in Supplementary Fig. 17b, IMI does not require the existence of electrons bound near defects. The strong electric field can excite the electrons from the valence to the conduction band and then accelerate the carriers so that impact ionization liberates additional carriers which in turn can be accelerated to induce further IMI, etc., in a cascading effect. Therefore, we consider 10^{14} cm^{-2} as a lower limit to the electron-hole pair density produced by THz excitation, and far higher densities are possible. Both PF and IMI processes scale approximately exponentially with the field strength. The ensuing phase switching when a sufficient carrier density is reached is also highly nonlinear, hence leading to the threshold behavior observed experimentally.

In addition, we remark that THz pulses are well known to limit heating effects in semiconductors compared to optical pulses. We estimate the energy deposition imparted to the sample by using the estimated carrier density and assuming each carrier is accelerated to reach the saturation velocity ($\sim 10^7$ cm/s) (ref.[34]). The transient temperature rise induced by THz fields can be qualitatively estimated by assuming all the carrier kinetic energies turning into heat, as given by $\Delta T = \frac{1}{2} m_e v_s^2 \cdot n/(C\rho d)$. Here, m_e is electron mass, v_s is saturation velocity, n is carrier density, C is the heat capacity, ρ is mass density, d is sample thickness, and we use the estimated carrier density $n \sim 10^{14}$–10^{15} cm^{-2}. Thus, our THz

fields can only heat up the sample by about 1–20 K.

If we assume that after the THz excitation there are possible field-emitted electrons trapped at the h-BN/fused silica interface, then the $MoTe_2$ can be charged with the same amount of holes. Although the whole system is still charge neutral, the spatial separation of electrons and holes is effectively charging the $MoTe_2$ layers. Also, we need to note that the generated electrons and holes may not be bound. The instantaneous field acceleration effect persists for approximately 500 fs. Based on the assumption that the carriers achieve their saturation speed, the maximal separation distance is estimated to be 100 nm. Such carrier separation may manifest at the sample center if there is a significant variation in electric field resulting from local enhancement or in the presence of trap states[36]. Therefore, we conclude that the driving mechanism for the phase transition can be due to charge doping and/or neutral carrier redistribution.

Supplementary Note 6. Theoretical calculations of phase transition barrier and energy landscape

We performed density functional theory (DFT) calculations using the VASP code[37] with generalized gradient approximation – Perdew-Burke-Ernzerhof (GGA-PBE) exchange-correlation functional[38]. The kinetic energy cutoff was 350 eV for the plane-wave basis sets. The lattice constants of $2H$ and $1T'$ phases in the neutral state were obtained via structural optimization. The vacuum region was more than 36 Å thick to decouple the neighboring slabs. A Γ-centered $11 \times 17 \times 1$ k-point mesh was used to sample the Brillouin zone. The climbing image nudged elastic band (CI-NEB) method[39] with 5 to 7 intermediate images was used to determine the activation barrier of the phase transition. In these barrier calculations, the in-plane lattice constants were all constrained to the value of the neutral state $2H$ phase, even for the charged state conditions in which the lattice tends to expand. This treatment of constant area was mainly due to difficulty of convergence when the lattice vectors were allowed to relax during CI-NEB calculations of charged slabs. It simulates situations where the lattice of monolayer $MoTe_2$ is strongly constrained by its interaction with substrates. The convergence threshold of force on each atom was 0.01 eV/Å. For charged state calculations, a compensating background charge was introduced to the slab, the same as the treatment in two earlier studies[4,40] of charge mediated phase transitions in MoS_2 and $MoTe_2$. We observe

that the activation energy decreases upon adding charge carriers, and the $1T'$ phase becomes thermodynamically more stable than the $2H/2H^*$ phase when the electron density exceeds 9×10^{14} cm^{-2}. Our DFT calculation also shows that the $2H$ to $2H^*$ phase transition is barrier-less even at a small doping level. It has also been reported that a transient current can drive the $2H$ to $2H^*$ phase transition in multilayer $MoTe_2$ encapsulated between two metals[41]. Therefore, it is possible that a single THz pulse could also initiate the $2H$-$2H^*$ phase transition.

Based on our estimate in Supplementary Note 5, our THz fields can strongly perturb the carrier distribution by generating electron-hole pairs, which retains the sample neutrality. Thus, we also investigate the effects of nonequilibrium electronic excitation for the $2H$-to-$1T'$ phase transition. The highly non-equilibrium THz-excited carrier distribution is difficult to treat theoretically since it deviates from the low-temperature ground state Fermi-Dirac distribution and populates more states at higher energies. Here, we qualitatively describe the THz-excited carrier distribution by a Fermi-Dirac distribution with temperature smearing. The smearing parameter in the computations can be adjusted to have a reasonable amount of population in the conduction band for easy approximate comparison with the experimental case. The smearing function in DFT replaces the step-function Fermi distribution at zero temperature by a smoother function in order to achieve smooth convergence of total energy computations. We have chosen a smearing temperature up to 0.5 eV, which causes a reasonable density of 4×10^{14} cm^{-2} of electrons to be excited from the valence band to the conduction band and approximates the THz-excitation case. From the DFT calculations for the free energy, we observe that the activation energy decreases with higher smearing temperature for the $2H$-to-$1T'$ transition, similar to the charge doping effect discussed above. The $2H^*$ phase was not observed with the temperature smearing effect, possibly due to the low smearing temperature[42].

As shown in Fig. 3c,d, two requirements are necessary for achieving a phase transition from $2H/2H^*$ to $1T'$. First, the energy of the $1T'$ phase needs to be lower than the $2H$ phase upon external excitation. Second, the activation barrier E_{act} between the two phases should be lowered compared to the equilibrium case. The relationship between the rate constant of the phase transition k and the activation barrier E_{barrier} is described by the Arrhenius

equation[43]

$$k = A \cdot \exp(-E_{\text{barrier}}/k_B T) \tag{S4}$$

where A is the attempt frequency. At room temperature $T = 300$ K, the rate constant k will increase by around twelve orders if E_{barrier} decreases from 1.66 eV to 0.91 eV. The qualitative trend of increasing the rate by many orders of magnitude matches with the experimental measurement, which confirms that the nonequilibrium carrier redistribution realized with our THz field can greatly speed up the phase transition.

Although the theoretical results are qualitatively consistent with experimental observations, there are several limitations that will have to be overcome in the future. First, the expansion and distortion of the in-plane lattice were suppressed for CI-NEB calculations, which can lead to overestimation of the activation energies. Second, for the charge doping calculation, the DFT energy may not be quite accurate due to the compensating background charge. A correction method[44] was proposed to describe the reaction energy more accurately. A comparative study[40] showed that the method did not change the overall picture. Third, we note that the free energy used here contains only the electronic contribution, without the phonon contributions. Additionally, the THz-driven nonequilibrium electronic distribution may deviate for some time from the Fermi-Dirac distribution, where the time is determined by the carrier-carrier scattering rate.

Supplementary Note 7. Theoretical calculations of electronic and topological properties along the phase transition pathway

In this section, we discuss the electronic structure calculations that we performed to evaluate the topological properties. Specifically, we estimated the Z_2 topological invariant to distinguish between the trivial insulator state and the quantum spin Hall state[45]. For $2H$ and $1T'$ structures, there are direct band gaps between the valence and conduction bands. This energy separation allowed us to investigate the topological property of the filled valence bands. In our Wannier models, this amounts to the filling of 28 electrons for the lowest bands. For $1T'$ bands, these filled bands are shown in Supplementary Fig. 23a. For inversion symmetric systems, the Fu-Kane parity formulation[46] can be utilized to efficiently

compute the topological invariant. This is computed by considering the inversion symmetry eigenvalues at the time-reversal invariant momenta points for the filled bands (Γ, X, Y, and R points in Supplementary Fig. 23c) The inversion symmetry center in real space is shown in Supplementary Fig. 23b. The symmetry transformations and the inversion symmetry eigenvalues were derived for the effective Wannier models constructed from full DFT calculations. The Z_2 topological invariant can be evaluated by

$$(-1)^{\nu} = \prod_{i} \delta_i, \quad \text{where } \delta_i = \prod_{m=1}^{N} \xi_{2m}(\Gamma_i), \tag{S5}$$

with $\xi_{2m}(\Gamma_i) = \pm 1$ the parity eigenvalues for the filled state pairs at TRIM point Γ_i as defined above, up to $(2N)$-th band. The non-trivial (trivial) Z_2 invariant is indicated by $\nu = 1$ ($\nu = 0$).

In the structural $2H/1T'$ transition for monolayer $MoTe_2$, the electronic structure also undergoes the transition from a trivial insulator phase to a non-trivial Z_2 topological phase[47]. The parity formulation above establishes the non-trivial topological character for $1T'$ phase. Here we are interested in the topological properties in between the structural transition. However, the intermediate structures do not respect inversion symmetry and the Fu-Kane formulation cannot be directly applied. To resolve the topological properties along the transition path, we employed the non-Abelian Berry connection calculation along the Wilson loop[48] and the numerical results for the transition path are shown in Supplementary Fig. 21. As in the figure, this method infers the non-trivial topological invariant from the "partner switching" of the Wannier centers during the time-reversal pumping. This can be read off by counting the number of crossing points on any horizontal line in the Wannier center evolution plot. An odd (even) number indicates a non-trivial (trivial) topological phase of the filled bands. These topological invariants can only change when the gap between conduction and valence bands collapses. In Supplementary Fig. 22, we also show the topological properties accompanied by the direct band gap evolution.

Supplementary Note 8. Absorption-based THz heating effect

Absorption calculation can be another way to estimate transient temperature increases. To assess the absorbed power, the crucial parameter is the penetration depth. Theoret-

ically, the penetration depth is infinite in $2H$-$MoTe_2$ since it has a much larger bandgap (∼1 eV) than the incident THz photon energy (∼3 meV), and direct or multi-photon absorption is negligible. The applied photon energy is also not resonant with any infrared-active phonons[22]. Therefore, negligible heating is present in the linear regime (low THz field strength). In reality, under intense THz illumination, nonlinear effects can emerge, and some photon energies are absorbed by the sample. However, we do not have exact knowledge of the penetration depth in this unexplored non-resonant excitation regime. Thus, we analyzed literature data[49] that have experimentally measured the penetration depth in GaAs at THz range (step 1a). Then, we performed the Keldysh simulation of strong-field ionization to translate the GaAs results to $2H$-$MoTe_2$ (step 1b). The results of the two-step estimate yields 6 μm as the penetration depth of $MoTe_2$ in the field strength we used. We will discuss the two steps separately below:

Step (1a): In Ref.[49], the nonlinear THz absorption was measured in bulk GaAs (500-μm thick) patterned with 200-nm-gap structures. Large nonlinear THz absorption arises mostly in the confined region of the nanometer-scale layer, so despite the large actual thickness of GaAs, the effective thickness of the sample deff was estimated to be similar to the gap size at 200 nm where the absorption is nonzero. The authors of that study applied in-gap field strength over 20 MV/cm and observed up to 35% change in absorption relative to the low-field regime due to nonlinear carrier generation. Based on the Beer-Lambert law, this observation means that the corresponding penetration depth d is around 464 nm at 20 MV/cm. Note that the field strength in the GaAs study is much larger than our case (5 MV/cm) mainly due to the smaller gap of metamaterials used in the GaAs study. Here, we would like to clarify the field strength of 5–27 MV/cm. Based on the simulation shown in Fig. 1c of the main text, we note that the majority of the sample experiences in-gap THz field at ∼5 MV/cm. The 27-MV/cm field strength only happens at a highly confined area/hotspot in the simulation that has negligible contribution in the probed volume, and is hence unlikely to contribute to our results.

Step (1b): Now, we need to translate the results of GaAs to our case of $2H$-$MoTe_2$. The bandgap of GaAs is ∼1.4 eV, larger than that of $2H$-$MoTe_2$ at ∼1 eV. Therefore, more carriers will be generated in $MoTe_2$ than GaAs under the same THz field strength. To find the equivalence between the two cases, we need to adopt a metric. Here we assume the ionization (carrier generation) rate is a good metric because the primary THz absorption

pathway is the ionization rate events, and this parameter is directly linked to the temperature increase described by the number of mobile carriers multiplied by their kinetic energy. Thus, we adopted the Keldysh formalism of strong-field ionization[50] to estimate ionization (electron and hole generation) rates Ω for materials with different bandgaps.

We calculated the ionization rate for GaAs with a bandgap of 1.4 eV and $MoTe_2$ with a bandgap of 1 eV. Since we applied ~5 MV/cm to $MoTe_2$, the simulation shows that the ionization rate for $MoTe_2$ under such field strengths is similar to those in GaAs under ~7.2 MV/cm. Thus, we estimate the absorption would be ~3.2% based on Ref[49]. This means the penetration depth is about 6.1 μm based on the Beer-Lambert formula. The absorbed THz power by the $MoTe_2$ sample with a thickness d (about 1 nm) can be estimated by the Beer-Lambert law. The transient temperature rise induced by the absorbed THz fields can be estimated by $\Delta T = \frac{1/2\varepsilon_0 F^2 c_0 \delta t(1-e^{-d/d_0})}{C\rho d}$, where F is the incident THz field, C is the heat capacity[51] at 200 $\mathrm{J{\cdot}kg^{-1}K^{-1}}$, ρ is the mass density at 7.7 $\mathrm{g/cm^3}$, d is the sample thickness at about 1 nm that is far smaller than d_0 and canceled, c_0 is the speed of light in vacuum, and $\delta t = 500$ fs is the THz pulse duration as shown in Supplementary Fig. 13, ε_0 is the vacuum permittivity. Therefore, the temperature increase is ~18 K, consistent with previous estimation in Supplementary Note 5.

Supplementary References

[1] Johnson, P. B. & Christy, R. W. Optical constants of the noble metals. *Phys. Rev. B* **6**, 4370–4379 (1972).

[2] Naftaly, M. & Miles, R. E. Terahertz time-domain spectroscopy for material characterization. *Proc. IEEE* **95**, 1658–1665 (2007).

[3] Laturia, A., Van de Put, M. L. & Vandenberghe, W. G. Dielectric properties of hexagonal boron nitride and transition metal dichalcogenides: from monolayer to bulk. *npj 2D Mater. Appl.* **2**, 6 (2018).

[4] Gao, P., Wang, L., Zhang, Y., Huang, Y. & Liu, K. Atomic-scale probing of the dynamics of sodium transport and intercalation-induced phase transformations in MoS_2. *ACS Nano* **9**, 11296–11301 (2015).

[5] Wang, L., Xu, Z., Wang, W. & Bai, X. Atomic mechanism of dynamic electrochemical lithiation processes of MoS_2 nanosheets. *J. Am. Chem. Soc.* **136**, 6693–6697 (2014).

[6] Meng, L., Ma, Y., Si, K., Xu, S., Wang, J. *et al.* Recent advances of phase engineering in group VI transition metal dichalcogenides. *Tungsten* **1**, 46–58 (2019).

[7] Heising, J. & Kanatzidis, M. G. Exfoliated and restacked MoS_2 and WS_2: Ionic or neutral species? Encapsulation and ordering of hard electropositive cations. *J. Am. Chem. Soc.* **121**, 11720–11732 (1999).

[8] Zhou, L., Zubair, A., Wang, Z., Zhang, X., Ouyang, F. *et al.* Synthesis of high-quality large-area homogenous $1T'$ $MoTe_2$ from chemical vapor deposition. *Adv. Mater.* **28**, 9526–9531 (2016).

[9] Chen, S.-Y., Naylor, C. H., Goldstein, T., Johnson, A. T. C. & Yan, J. Intrinsic phonon bands in high-quality monolayer T′ molybdenum ditelluride. *ACS Nano* **11**, 814–820 (2017).

[10] Si, C., Choe, D., Xie, W., Wang, H., Sun, Z. *et al.* Photoinduced vacancy ordering and phase transition in $MoTe_2$. *Nano Lett.* **19**, 3612–3617 (2019).

[11] Cho, S., Kim, S., Kim, J. H., Zhao, J., Seok, J. *et al.* Phase patterning for ohmic homojunction contact in $MoTe_2$. *Science* **349**, 625–628 (2015).

[12] Song, Y., Tian, R., Yang, J., Yin, R., Zhao, J. *et al.* Second harmonic generation in atomically thin $MoTe_2$. *Adv. Opt. Mater.* **6**, 1701334 (2018).

[13] Tan, Y., Luo, F., Zhu, M., Xu, X., Ye, Y. *et al.* Controllable 2H-to-1T′ phase transition in

few-layer $MoTe_2$. *Nanoscale* **10**, 19964–19971 (2018).

[14] Kim, S., Kim, J. H., Kim, D., Hwang, G., Baik, J. *et al.* Post-patterning of an electronic homojunction in atomically thin monoclinic $MoTe_2$. *2D Materials* **4**, 024004 (2017).

[15] Chapman, H. N., Fromme, P., Barty, A., White, T. A., Kirian, R. A. *et al.* Femtosecond X-ray protein nanocrystallography. *Nature* **470**, 73–77 (2011).

[16] Seibert, M. M., Ekeberg, T., Maia, F. R. N. C., Svenda, M., Andreasson, J. *et al.* Single mimivirus particles intercepted and imaged with an X-ray laser. *Nature* **470**, 78–81 (2011).

[17] Boutet, S., Lomb, L., Williams, G. J., Barends, T. R. M., Aquila, A. *et al.* High-resolution protein structure determination by serial femtosecond crystallography. *Science* **337**, 362–364 (2012).

[18] Redecke, L., Nass, K., DePonte, D. P., White, T. A., Rehders, D. *et al.* Natively inhibited Trypanosoma brucei Cathepsin B structure determined by using an X-ray laser. *Science* **339**, 227–230 (2013).

[19] Aquila, A., Hunter, M. S., Doak, R. B., Kirian, R. A., Fromme, P. *et al.* Time-resolved protein nanocrystallography using an X-ray free-electron laser. *Opt. Express* **20**, 2706–2716 (2012).

[20] Tenboer, J., Basu, S., Zatsepin, N., Pande, K., Milathianaki, D. *et al.* Time-resolved serial crystallography captures high-resolution intermediates of photoactive yellow protein. *Science* **346**, 1242–1246 (2014).

[21] Peng, B., Zhang, H., Chen, W., Hou, B., Qiu, Z.-J. *et al.* Sub-picosecond photo-induced displacive phase transition in two-dimensional $MoTe_2$. *npj 2D Mater. Appl.* **4** (2020).

[22] Froehlicher, G., Lorchat, E., Fernique, F., Joshi, C., Molina-Sánchez, A. *et al.* Unified description of the optical phonon modes in N-layer $MoTe_2$. *Nano Lett.* **15**, 6481–6489 (2015).

[23] Novoselov, K. S., Jiang, D., Schedin, F., Booth, T. J., Khotkevich, V. V. *et al.* Two-dimensional atomic crystals. *Proceedings of the National Academy of Sciences* **102**, 10451–10453 (2005).

[24] Liu, M., Hwang, H. Y., Tao, H., Strikwerda, A. C., Fan, K. *et al.* Terahertz-field-induced insulator-to-metal transition in vanadium dioxide metamaterial. *Nature* **487**, 345–348 (2012).

[25] Zhu, H., Wang, Q., Cheng, L., Addou, R., Kim, J. *et al.* Defects and surface structural stability of $MoTe_2$ under vacuum annealing. *ACS Nano* **11**, 11005–11014 (2017).

[26] Hong, J., Hu, Z., Probert, M., Li, K., Lv, D. *et al.* Exploring atomic defects in molybdenum disulphide monolayers. *Nat. Commun.* **6**, 6293 (2015).

[27] Qiu, H., Xu, T., Wang, Z., Ren, W., Nan, H. *et al.* Hopping transport through defect-induced localized states in molybdenum disulphide. *Nat. Commun.* **4**, 2642 (2013).

[28] Pradhan, N. R., Rhodes, D., Feng, S., Xin, Y., Memaran, S. *et al.* Field-effect transistors based on few-layered α-$MoTe_2$. *ACS Nano* **8**, 5911–5920 (2014).

[29] Lin, Y.-F., Xu, Y., Wang, S.-T., Li, S.-L., Yamamoto, M. *et al.* Ambipolar $MoTe_2$ transistors and their applications in logic circuits. *Adv. Mater.* **26**, 3263–3269 (2014).

[30] Bie, Y.-Q., Grosso, G., Heuck, M., Furchi, M. M., Cao, Y. *et al.* A $MoTe_2$-based light-emitting diode and photodetector for silicon photonic integrated circuits. *Nat. Nanotechnol.* **12**, 1124–1129 (2017).

[31] Yeargan, J. R. & Taylor, H. L. The Poole-Frenkel effect with compensation present. *J. Appl. Phys.* **39**, 5600–5604 (1968).

[32] Simmons, J. G. Poole-Frenkel effect and Schottky effect in metal-insulator-metal systems. *Phys. Rev.* **155**, 657–660 (1967).

[33] Pein, B. C., Chang, W., Hwang, H. Y., Scherer, J., Coropceanu, I. *et al.* Terahertz-driven luminescence and colossal Stark effect in CdSe–CdS colloidal quantum dots. *Nano Lett.* **17**, 5375–5380 (2017).

[34] Sze, S. M. & Ng, K. K. *Physics of Semiconductor Devices* (John Wiley & Sons, Ltd, Hoboken, New Jersey, 2007).

[35] Fan, K., Hwang, H. Y., Liu, M., Strikwerda, A. C., Sternbach, A. *et al.* Nonlinear terahertz metamaterials via field-enhanced carrier dynamics in GaAs. *Phys. Rev. Lett.* **110**, 217404 (2013).

[36] Ju, L., Velasco, J., Huang, E., Kahn, S., Nosiglia, C. *et al.* Photoinduced doping in heterostructures of graphene and boron nitride. *Nat. Nanotechnol.* **9**, 348–352 (2014).

[37] Kresse, G. & Furthmüller, J. Efficient iterative schemes for *ab initio* total-energy calculations using a plane-wave basis set. *Phys. Rev. B* **54**, 11169–11186 (1996).

[38] Perdew, J. P., Burke, K. & Ernzerhof, M. Generalized gradient approximation made simple. *Phys. Rev. Lett.* **77**, 3865–3868 (1996).

[39] Henkelman, G., Uberuaga, B. P. & Jónsson, H. A climbing image nudged elastic band method for finding saddle points and minimum energy paths. *J. Chem. Phys.* **113**, 9901–9904 (2000).

[40] Zhang, C., KC, S., Nie, Y., Liang, C., Vandenberghe, W. G. *et al.* Charge mediated reversible metal–insulator transition in monolayer $MoTe_2$ and $W_xMo_{1-x}Te_2$ alloy. *ACS Nano* **10**, 7370–

7375 (2016).

[41] Zhang, F., Zhang, H., Krylyuk, S., Milligan, C. A., Zhu, Y. *et al.* Electric-field induced structural transition in vertical $MoTe_2$- and $Mo_{1-x}W_xTe_2$-based resistive memories. *Nat. Mater.* **18**, 55–61 (2019).

[42] Kolobov, A. V., Fons, P. & Tominaga, J. Electronic excitation-induced semiconductor-to-metal transition in monolayer $MoTe_2$. *Phys. Rev. B* **94**, 094114 (2016).

[43] Zhang, H. & Banfield, J. F. Size dependence of the kinetic rate constant for phase transformation in TiO_2 nanoparticles. *Chem. Mater.* **17**, 3421–3425 (2005).

[44] Li, Y., Duerloo, K.-A. N., Wauson, K. & Reed, E. J. Structural semiconductor-to-semimetal phase transition in two-dimensional materials induced by electrostatic gating. *Nat. Commun.* **7**, 10671 (2016).

[45] Hasan, M. Z. & Kane, C. L. Colloquium: Topological insulators. *Rev. Mod. Phys.* **82**, 3045–3067 (2010).

[46] Fu, L. & Kane, C. L. Topological insulators with inversion symmetry. *Phys. Rev. B* **76**, 045302 (2007).

[47] Qian, X., Liu, J., Fu, L. & Li, J. Quantum spin Hall effect in two-dimensional transition metal dichalcogenides. *Science* **346**, 1344–1347 (2014).

[48] Yu, R., Qi, X. L., Bernevig, A., Fang, Z. & Dai, X. Equivalent expression of $\mathbb{Z}_2$ topological invariant for band insulators using the non-Abelian Berry connection. *Phys. Rev. B* **84**, 075119 (2011).

[49] Jeong, Y.-G., Paul, M. J., Kim, S.-H., Yee, K.-J., Kim, D.-S. *et al.* Large enhancement of nonlinear terahertz absorption in intrinsic GaAs by plasmonic nano antennas. *Applied Physics Letters* **103**, 171109 (2013).

[50] Keldysh, L. V. Ionization in the field of a strong electromagnetic wave. *Sov. Phys. JETP* **20**, 1307 (1964).

[51] Kobayashi, A., Takano, Y. & Demura, S. Observation of the specific heat jump in the Se-substituted $MoTe_2$ single crystals. *Materials* **15** (2022).

[52] Xi, X., Zhao, L., Wang, Z., Berger, H., Forró, L. *et al.* Strongly enhanced charge-density-wave order in monolayer $NbSe_2$. *Nat. Nanotechnol.* **10**, 765–769 (2015).

[53] Song, Q., Wang, H., Pan, X., Xu, X., Wang, Y. *et al.* Anomalous in-plane anisotropic raman response of monoclinic semimetal 1T′-$MoTe_2$. *Sci. Rep.* **7**, 1758 (2017).

[54] Li, Y., Rao, Y., Mak, K. F., You, Y., Wang, S. *et al.* Probing symmetry properties of few-layer MoS_2 and h-BN by optical second-harmonic generation. *Nano Lett.* **13**, 3329–3333 (2013).

[55] Bie, Y.-Q., Zong, A., Wang, X., Jarillo-Herrero, P. & Gedik, N. A versatile sample fabrication method for ultrafast electron diffraction. *Ultramicroscopy* **230**, 113389 (2021).

Supplementary Figures

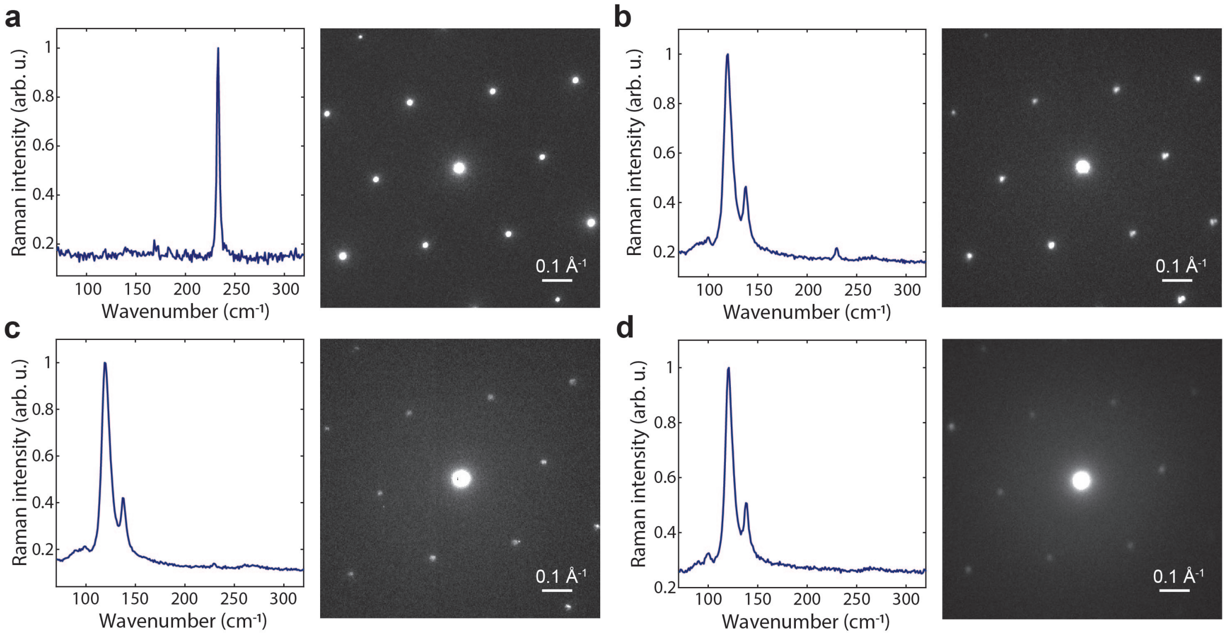

Supplementary Figure 1. **Sample damage induced by mid-infrared (MIR) excitations in multilayer $MoTe_2$.** **a**, Raman spectrum (left) and electron diffraction pattern (right) of pristine multilayer $MoTe_2$ before MIR irradiation (5.5 μm, $\sim$ 200 fs). **b–d**, Raman spectrum (left) and electron diffraction pattern (right) of different regions of the multilayer $MoTe_2$ sample after MIR irradiation. Two new peaks at 121 cm^{-1} and 142 cm^{-1} show up in Raman spectrum, while no new peaks show up in the diffraction pattern. The diffracted electron beam strengths correlate with the $2H$ Raman peak intensities at 235 cm^{-1}, indicating the diffraction signal originates from the residual $2H$ phase. The observation further confirms the Te-metalloid origin of the two new Raman peaks and sample damage induced by MIR excitation.

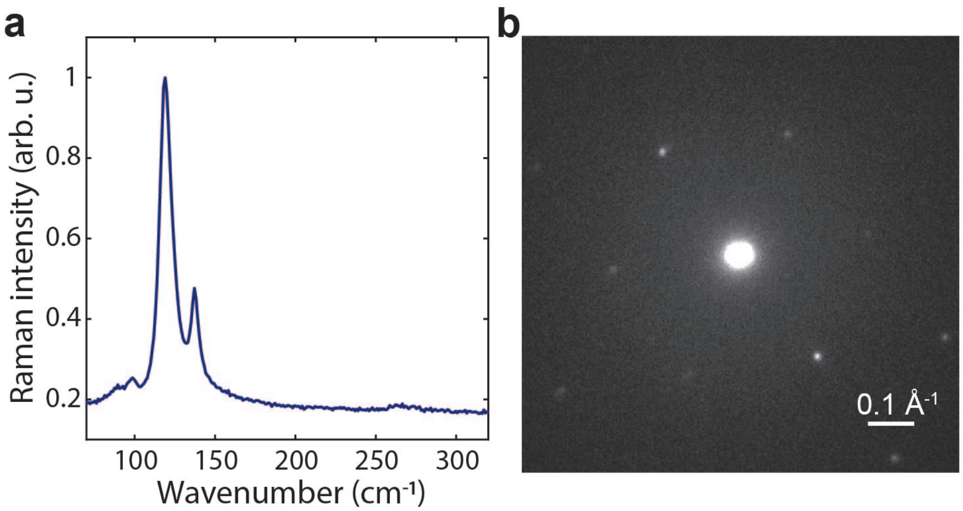

Supplementary Figure 2. **Sample damage induced by near-infrared (NIR) excitations in multilayer $\mathbf{MoTe_2}$.** Raman spectrum (**a**) and electron diffraction pattern (**b**) of pristine 2H $MoTe_2$ after NIR irradiation (800 nm, ~ 35 fs). Two new peaks at 121 cm^{-1} and 142 cm^{-1} show up in the Raman spectrum, while no new peaks show up in the diffraction pattern. The observation further confirms the Te-metalloid origin of the two new Raman peaks and sample damage induced by NIR excitation.

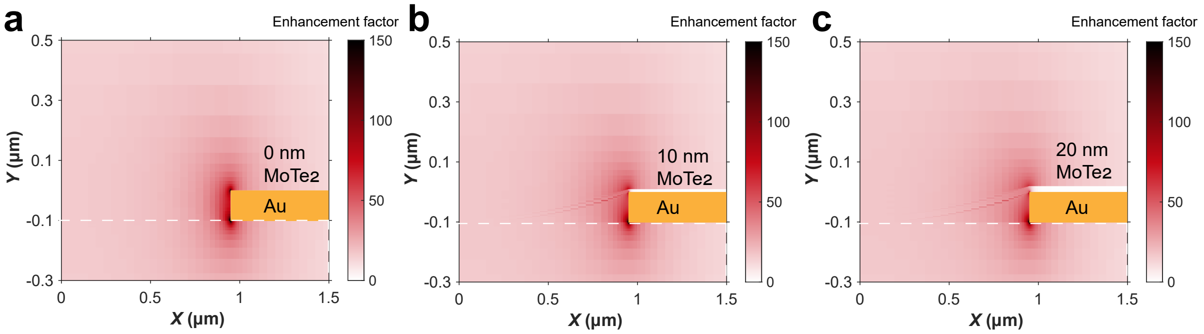

Supplementary Figure 3. **Numerical calculation of field strengths of the THz enhancement structure with different thicknesses of $MoTe_2$.** Different thicknesses of $2H$-$MoTe_2$ were used in the simulation, where 0 nm represents a bare Au slit without any $MoTe_2$ sample (panel **a**). Under the dashed line is the fused silica substrate region and the color bar represents the field enhancement factor. Only the right side of the Au gap is plotted, which shows decreasing trend of enhancement as the sample thickness increases. The enhancement remains high even with 20-nm $MoTe_2$, thereby indicating that the transition may still happen for thicker samples.

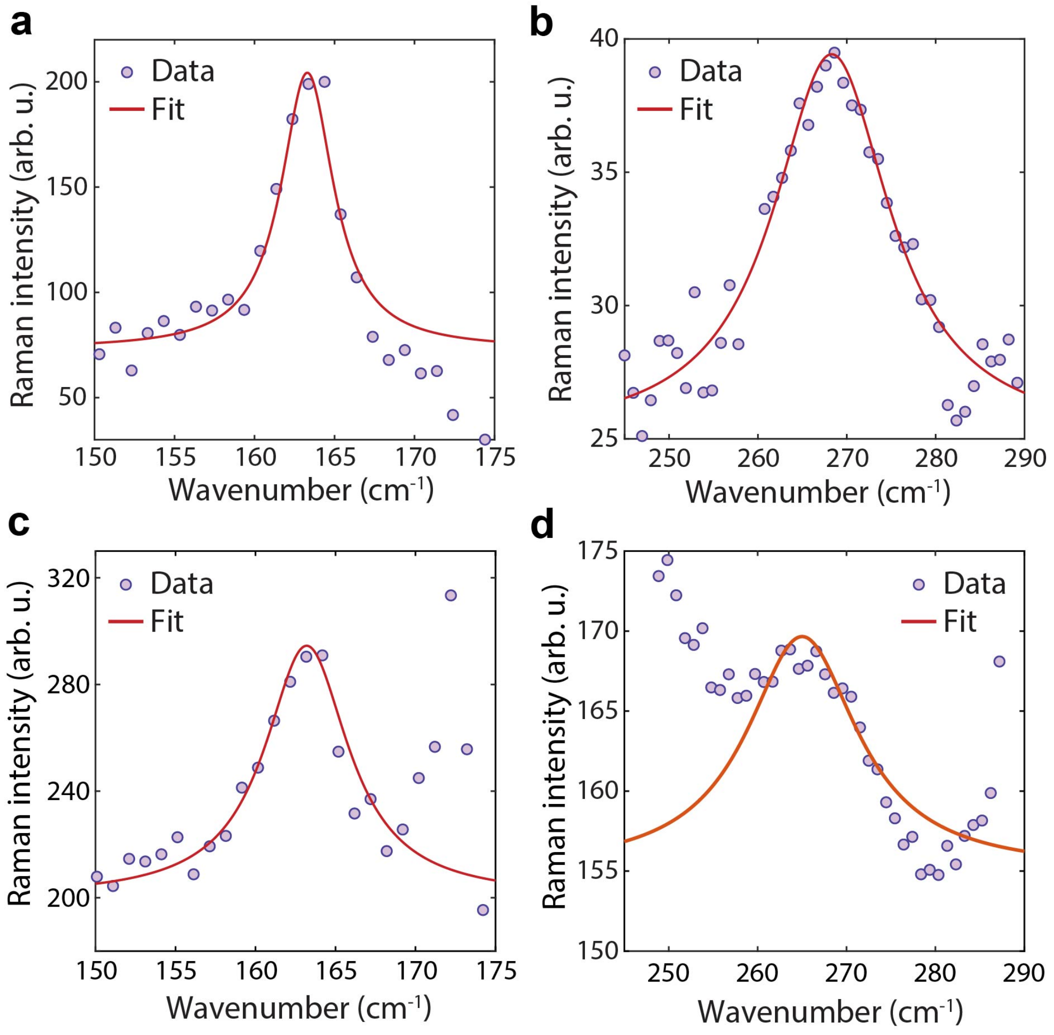

Supplementary Figure 4. **Lorentzian function fits for Raman spectra after THz irradiation.** In order to determine the Raman peak positions and linewidths in the THz-driven $1T'$ phase in monolayer and bilayer $MoTe_2$, the Raman peaks are fitted with a Lorentzian function: $I = I_0 + \frac{2A}{\pi}\frac{\Gamma}{4(x-x_c)^2+\Gamma^2}$, which peaks above the background signal intensity I_0 at frequency x_c with linewidth Γ and amplitude A[52]. **a**,**b**, Raman spectrum for monolayer $MoTe_2$ after THz irradiation, near $1T'$ A_g modes at frequencies $x_c = 163.3$ cm^{-1} (linewidth $\Gamma = 4$ cm^{-1}) and 268.6 cm^{-1} ($\Gamma = 16$ cm^{-1}) determined by single-Lorentzian fits. These two emergent peaks agree well with the Raman spectrum of $1T'$ monolayer $MoTe_2$ grown by chemical vapor deposition[53]. **c**,**d**, Raman spectrum for bilayer $MoTe_2$ after THz irradiation, near $1T'$ A_g modes at frequencies $x_c = 163$ cm^{-1} ($\Gamma = 6.4$ cm^{-1}) and 266 cm^{-1} ($\Gamma = 15$ cm^{-1}, fit approximately to the high-frequency side). The $2H$-to-$1T'$ phase transition is not complete in bilayer $MoTe_2$, as evidenced by the remaining $2H$ A_{1g} peak intensity at 171.5 cm^{-1}.

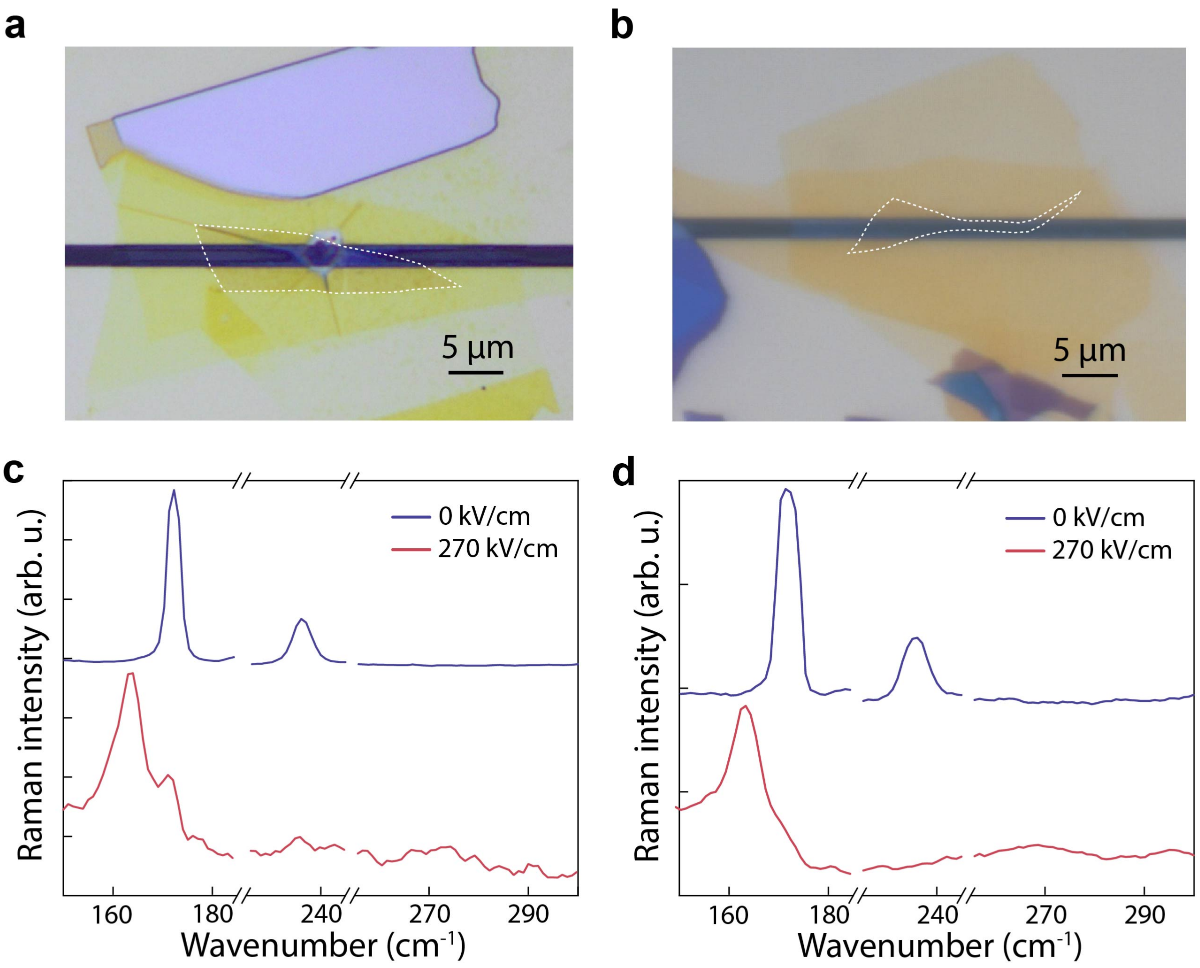

Supplementary Figure 5. **Additional monolayer $MoTe_2$ samples before and after THz irradiation.** **a**, The h-BN-encapsulated monolayer $MoTe_2$ sample No. 1 on a 2-μm insulating gap. The white dashed dots outline the shape of the monolayer $MoTe_2$. A dust particle was encapsulated and caused the protrusion in the middle. This did not measurably affect the behavior of the $MoTe_2$ at other areas in the gap. **b**, The h-BN-encapsulated monolayer $MoTe_2$ sample No. 2 on a 1.7-μm insulating gap is outlined with white dots. **c**,**d**, Raman spectra of samples No. 1 and No. 2 in the gaps before and after irradiation by THz fields with free-space amplitude of 270 kV/cm.

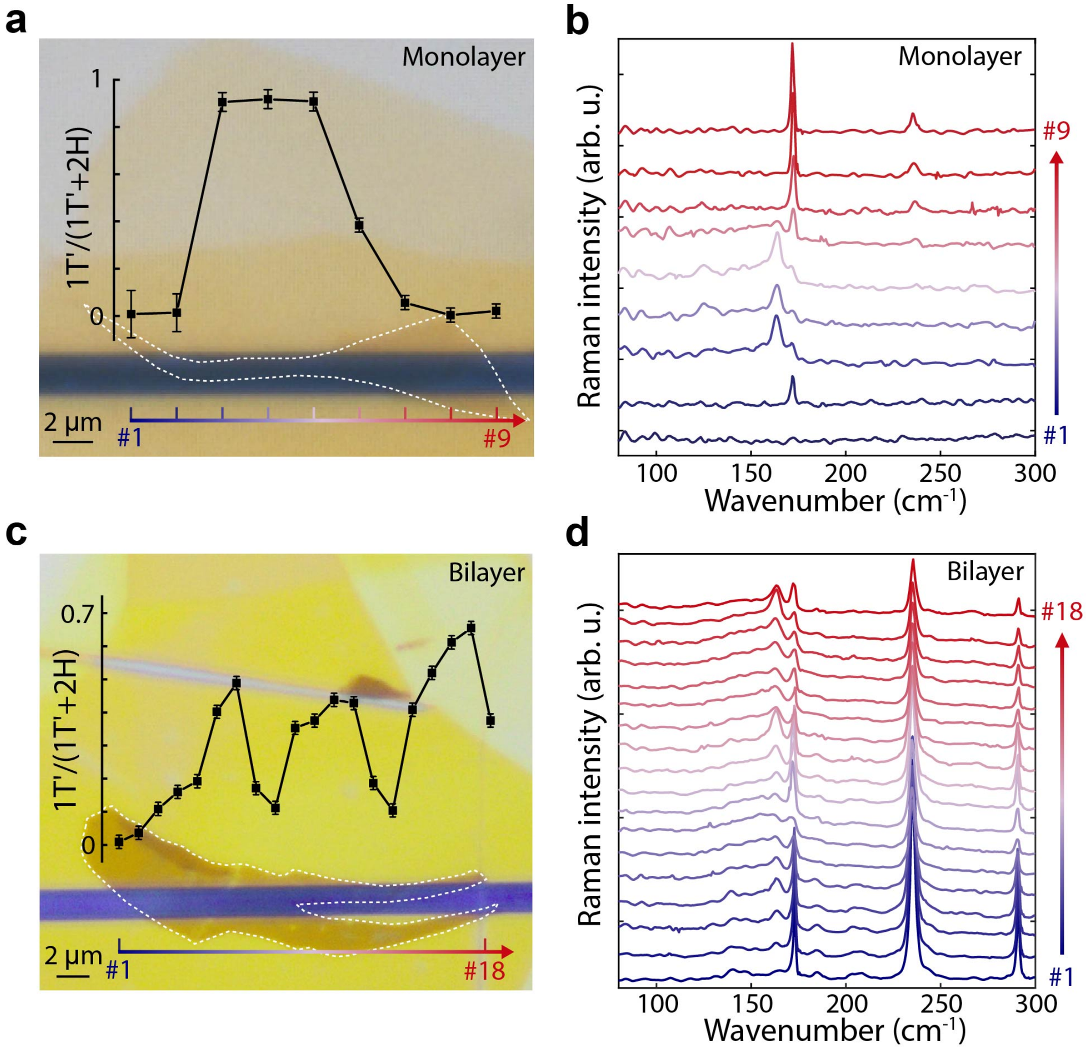

Supplementary Figure 6. **Raman line scans of monolayer and bilayer $MoTe_2$ samples after THz irradiation.** The laser beam was focused in the center of the gap and the sample was translated along the gap. **a**, Optical image of monolayer $MoTe_2$ after THz irradiation. The bottom marker (vertically offset for clear observation of the sample in the gap) shows the horizontal positions at which the spectra were recorded. The intensity ratio of Raman peaks at 163 cm^{-1} and 171 cm^{-1} is used to represent the conversion ratio $1T'/(1T'+2H)$. **b**, Raman spectra from the line scan in **a**. Some areas of monolayer $MoTe_2$ completely convert from $2H$ to $1T'$ while other areas have a smaller conversion ratio. There are several reasons that may cause an inhomogeneous response. First, the THz field is a fringe field with spatial variation across the gap rather than uniform enhancement. Second, the probing beam spot size is about 1 μm in diameter, which is close to the gap size. The inhomogeneity of the field enhancement at the gap edge means that the sample in our probing area experiences different levels of THz field strength irradiation[33]. **c**, Optical image and the conversion ratio of bilayer $MoTe_2$ after THz irradiation. **d**, Raman spectra from the line scan in **c**. For bilayer $MoTe_2$, the conversion efficiency is between 0 and 0.7 and no area is completely converted to the $1T'$ phase. For $1T'$ $MoTe_2$, the Raman signature at around 268 cm^{-1} is much weaker than the A_g peak at around 163 cm^{-1}, and can only be observed in some areas of the sample in the gap, as shown in **b** and **d**.

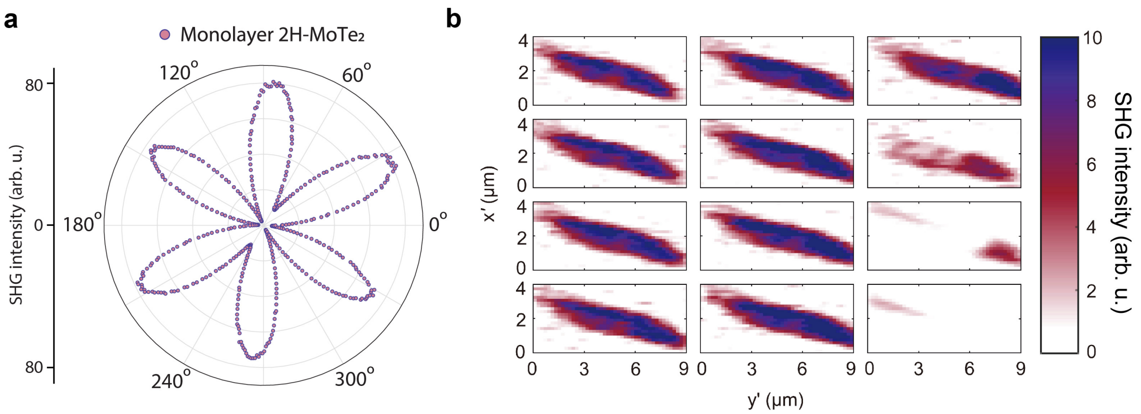

Supplementary Figure 7. **Polarization dependence and field dependence of SHG in monolayer $MoTe_2$.** **a**, Polar plot of the SHG intensity from monolayer $MoTe_2$ as a function of the crystal's azimuthal angle θ relative to the incident light polarization direction. The optical image of the sample is shown in Fig. 1d. The dependence of SHG intensity on the incident polarization angle exhibits six-fold rotational symmetry, in accordance with the predicted angular variation of SHG in hexagonal phase 2D materials[54]. **b**, SHG microscopy images of monolayer $MoTe_2$ recorded after irradiation with single THz pulses with increasing field strength from 0 kV/cm to 270 kV/cm with an increment of about 20–30 kV/cm. The second harmonic signal mostly disappears after irradiation with a single THz pulse with a field strength higher than 210 kV/cm.

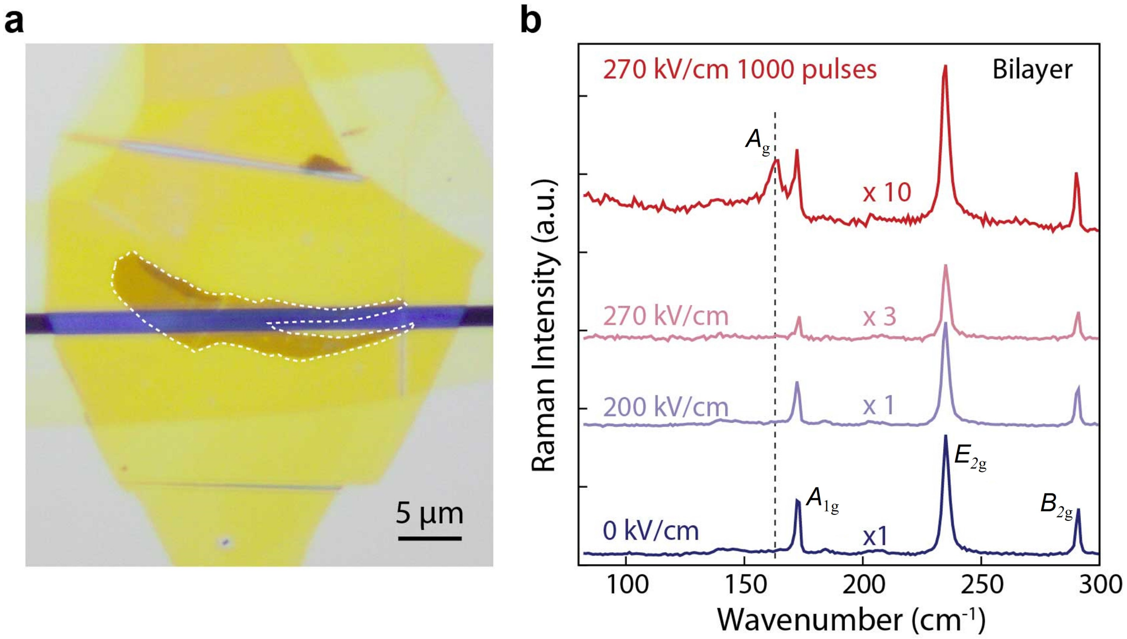

Supplementary Figure 8. **Bilayer $MoTe_2$ samples before and after THz irradiation with increasing field strengths.** **a**, The h-BN-encapsulated bilayer $MoTe_2$ sample. The white dashed dots outline the shape of the bilayer $MoTe_2$. **b**, Raman spectra of the bilayer sample in the gap before and after irradiation by THz fields with increasing strengths.

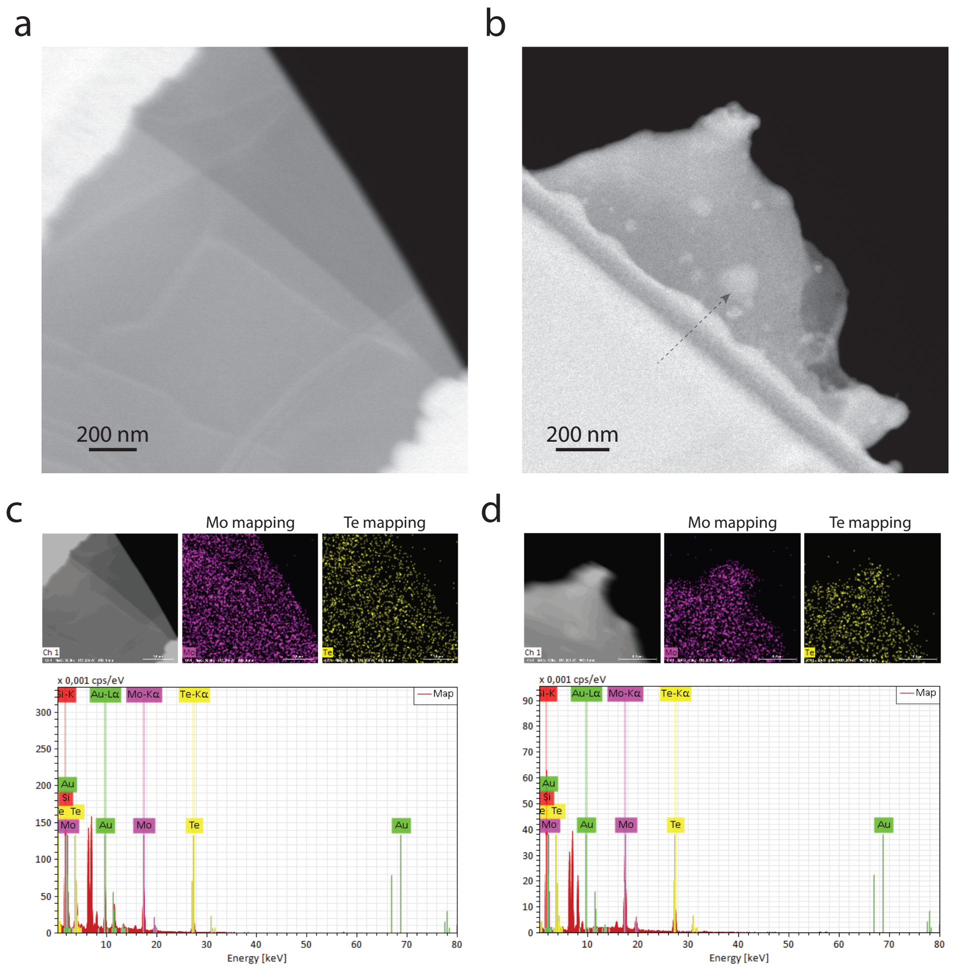

Supplementary Figure 9. **Experimental results of energy-dispersive X-ray spectroscopy (EDS) mapping under the scanning TEM mode.** **a**, TEM images of the pristine multilayer 2H-$MoTe_2$ flake suspended on TEM grids. **b**, Converted multilayer 1T'-$MoTe_2$ flake suspended on TEM grids. The bubble-like contrast in the image (marked by the arrow) is related to gas bubbles formed during the flake transfer process[55]. **c**, EDS Mo and Te mapping (top) and EDS spectrum (bottom) of the pristine 2H-$MoTe_2$. **d**, Similar to panel **c** but for the converted 1T'-$MoTe_2$.

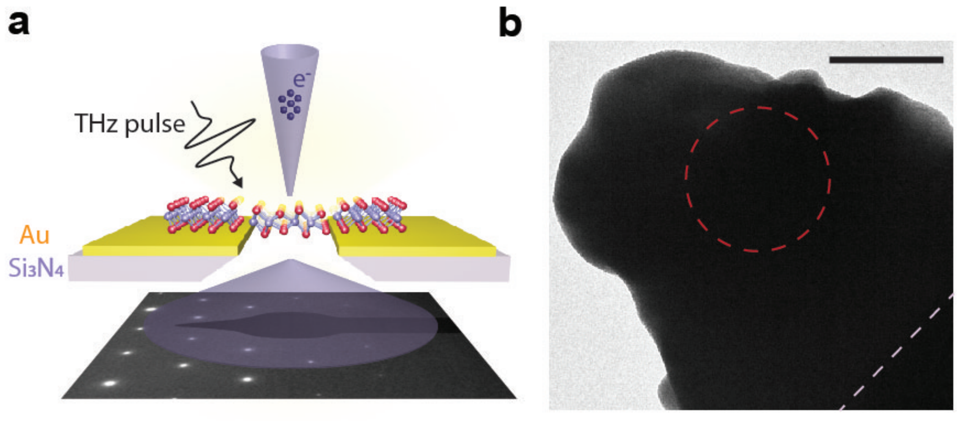

Supplementary Figure 10. **Experimental details of transmission electron microscope (TEM) measurements.** **a**, Schematic illustration of TEM measurements for sample characterization upon THz excitation. **b**, The TEM image of the sample after THz irradiation. The red dashed circle shows the electron beam size on the sample to generate the electron diffraction pattern. The purple dashed line marks the boundary of the free-standing sample, where parts of the sample at the bottom right corner are attached to a substrate. The scale bar represents 100 nm.

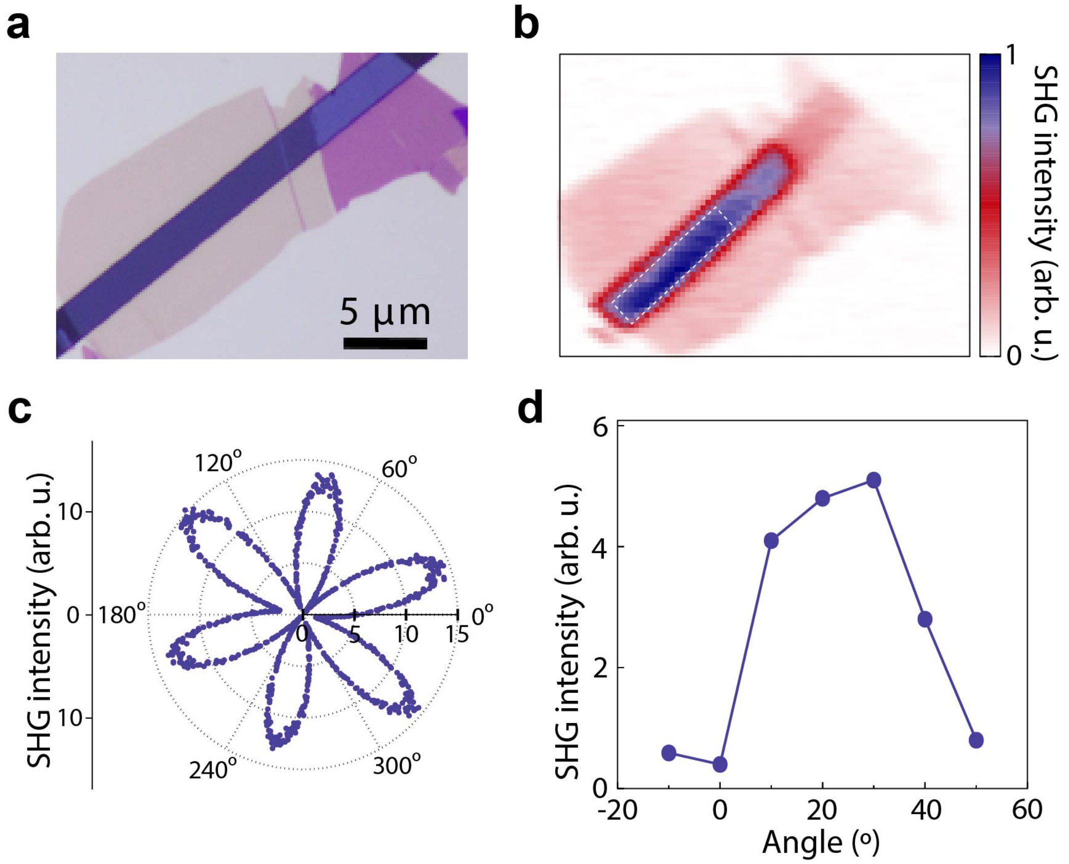

Supplementary Figure 11. **SHG characteristics of trilayer $MoTe_2$ samples for single-shot phase transition dynamics measurements.** **a**,**b**, Optical and SHG images of a typical trilayer $MoTe_2$ sample for single-shot ultrafast dynamic investigations. The white dashed area was selected to perform the single-shot measurement since it has the best uniformity. The SHG intensity variation inside the dashed line was within 5%. **c**, Polar plot of the SHG intensity from trilayer $MoTe_2$ as a function of the incident 800-nm beam (repetition rate 80 MHz) polarization angle. The incident pulse fluence is around 10 $\mu J/cm^2$, which is lower than the $MoTe_2$ optical damage threshold. The SHG radiation component detected is parallel to the polarization of the fundamental field. **d**, Polarization dependence of SHG intensity as a function of the 800-nm beam (single pulse) polarization angle. The incident pulse fluence is well above the optical damage threshold at around 20 mJ/cm^2. The SHG radiation component detected is parallel to the polarization of the fundamental field. Each data point was taken at a different sample position, with a different incident light polarization. The observation of one lobe of the six-fold symmetry confirms that the SHG is that of the crystal structure encountered by the incident pulses even though they damaged the sample.

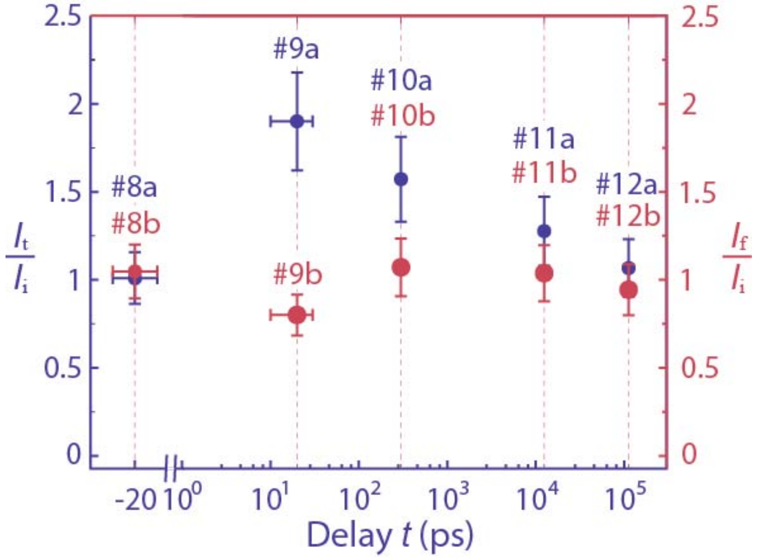

Supplementary Figure 12. **Additional THz-induced dynamics with below-threshold electric fields.** Similar single-shot pump-probe measurements on five bulk $2H$-$MoTe_2$ flakes. The free-space THz field strength was 150 kV/cm, below the polymorphic phase transition threshold. We used the same convention of labeling the data points as in Fig. 3b. The error analysis is provided in Supplementary Note 4

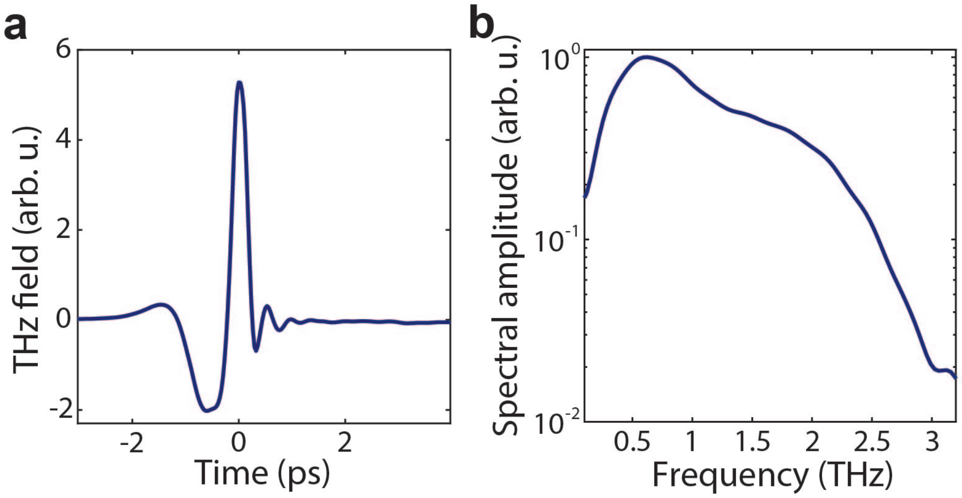

Supplementary Figure 13. **THz field and spectrum characteristics.** **a**, The temporal trace of the incident free-space single-cycle THz pulse. **b**, The THz spectrum is centered at around 0.5 THz and spans from 0.1 THz to 2.5 THz.

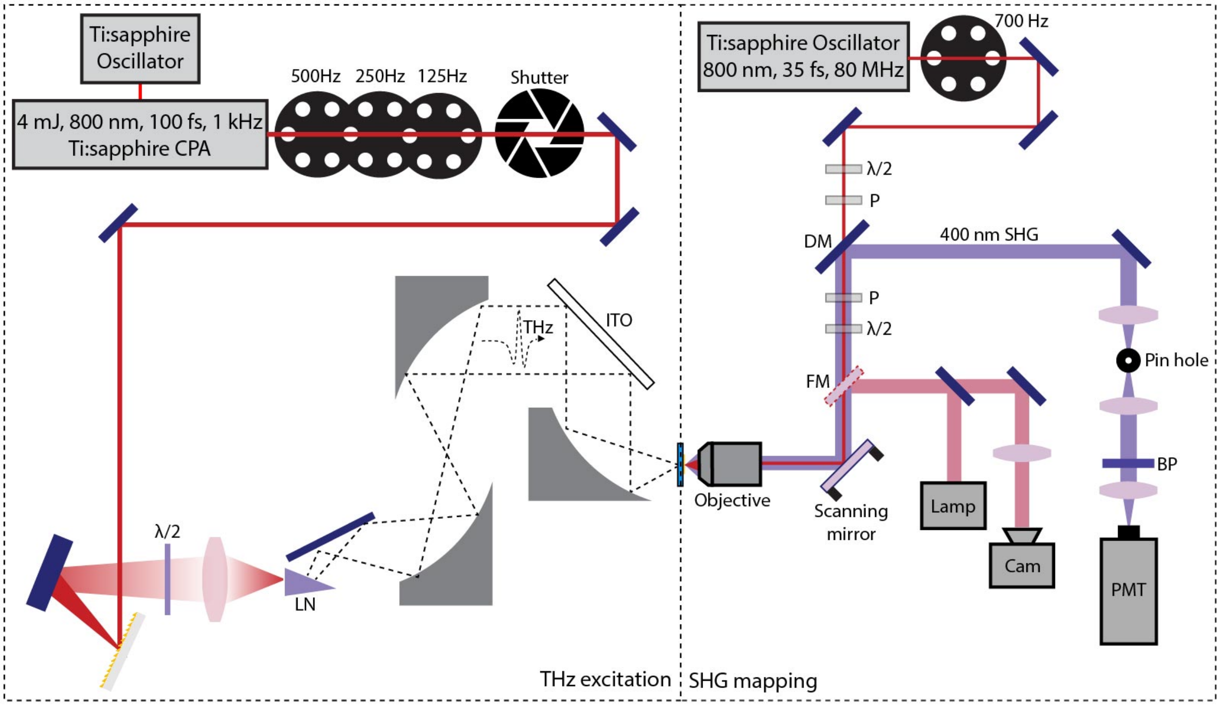

Supplementary Figure 14. **Schematic illustration of the setup used for *in situ* THz-SHG mapping measurements.** The left panel is for high-field THz pulse generation. The right panel is for *in situ* SHG mapping using an oscillator. LN: Lithium niobate crystal, ITO: Indium tin oxide window, P: Polarizer, $\lambda/2$: Half-wave plate, PH: Pinhole, BP: 400nm bandpass filter (40 nm bandwidth), PD: Photodiode, DM: Dichroic Mirror, FM: Flip mirror, Cam: camera, PMT: photomultiplier tube.

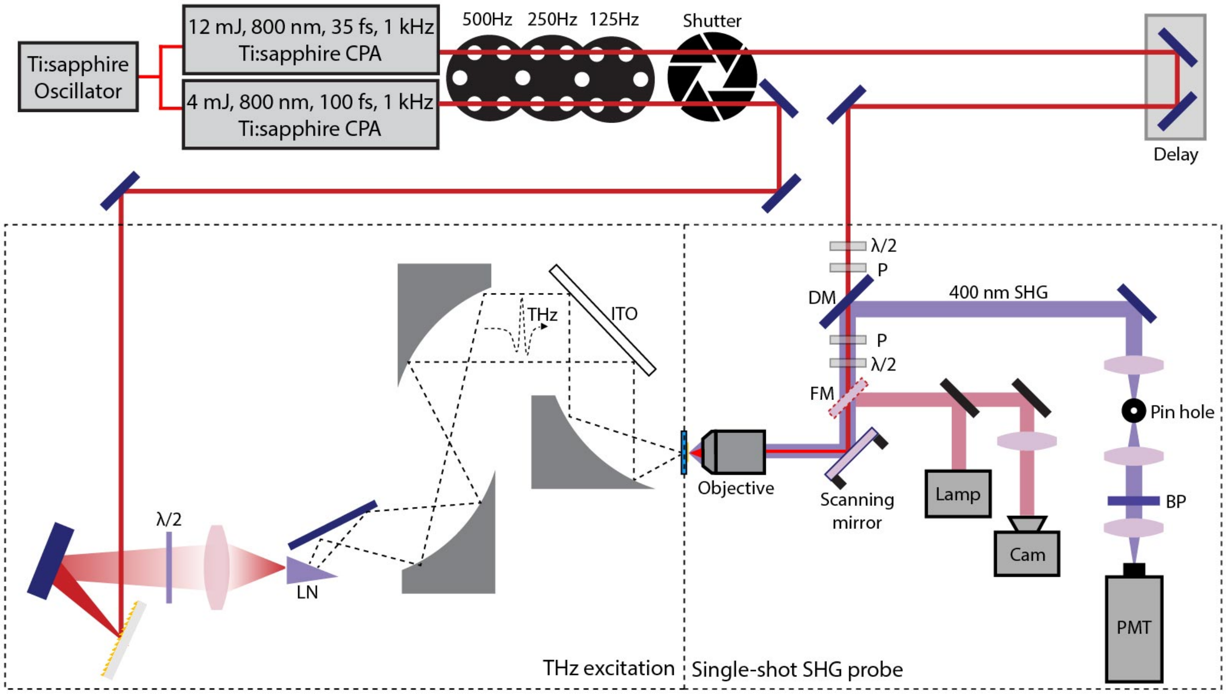

Supplementary Figure 15. **Schematic illustrations of single-shot THz pump and single-shot SHG probe microscopy.** Output pulses from the two amplifiers (1 kHz) were down-counted to 125 Hz by three successive choppers to allow enough temporal separation between pulses for a mechanical shutter to isolate single pulses. THz generation is the same as that shown in Supplementary Fig. 14. The single-shot SHG fundamental (800 nm) pulse is from a chirped pulse amplifier (CPA). The usage of two synchronized CPAs as pump and probe arms enables an arbitrary timing adjustment between the pump and probe arm. Fine-tuning (< 12.5 ns) of the pump-probe delay was realized by routing the beam via a translational stage. Longer delays were controlled electronically. The 12-mJ pulses from one of the CPAs were attenuated to around 20 mJ/cm^2 fluence at the sample when focused to a nearly diffraction-limited size (1.5 μm) with a 50× objective. This fluence was necessary for an adequate single-shot SHG signal/noise ratio, but it was typically well above the damage threshold of the $MoTe_2$ samples. LN: Lithium niobate crystal, ITO: Indium tin oxide window, P: Polarizer, $\lambda/2$: Half-wave plate, PH: Pinhole, BP: 400-nm bandpass filter (40 nm bandwidth), PD: photodiode, DM: dichroic mirror, FM: flip mirror, Cam: camera, PMT: photomultiplier tube.

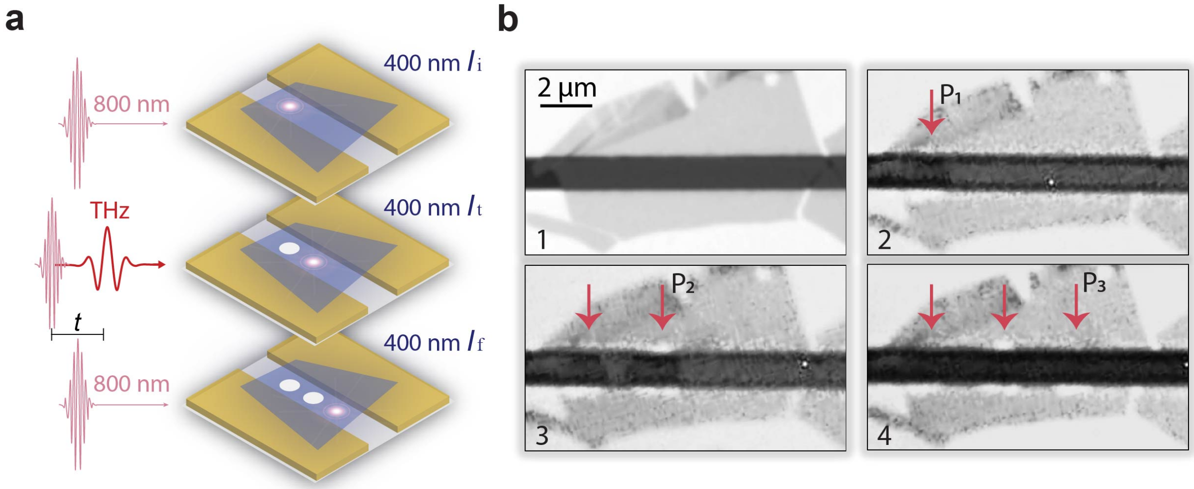

Supplementary Figure 16. **Experimental details of single-shot THz pump SHG probe measurements by sample replenishment. a**, Schematic illustration of single-shot SHG probe before and after a THz excitation pulse. Single-shot measurements were conducted with 800-nm pulses at three sample locations to record the SHG intensity from the sample in its initial state prior to the THz pulse (I_i), at a specified delay time after the THz pulse (I_t), and in its final state ~ 1 min after the THz pulse (I_f). The 35-fs, 800-nm probe pulses were focused to a spot size of ~ 1 μm in the center of the microslit gap, where the THz excitation fluence was about 20 mJ/cm^2. **b**, Optical images of a trilayer $MoTe_2$ sample used for SHG measurement. The pristine sample is shown in image 1 taken with a high resolution camera; images 2–4 were taken *in situ* with a low resolution camera. Image 2 was taken after measurement of SHG signal I_i by the first 800 nm pulse P_1. Image 3 was taken after another SHG signal I_i was measured by a second 800 nm pulse P_2; Image 4 was taken after THz excitation with 270 kV/cm field strength and measurement of SHG signal I_t by the third pulse P_3. The optically damaged regions are indicated by red arrows. The reduced optical reflectivity of the sample is apparent after THz irradiation which permanently removed the initial $2H$ phase of $MoTe_2$.

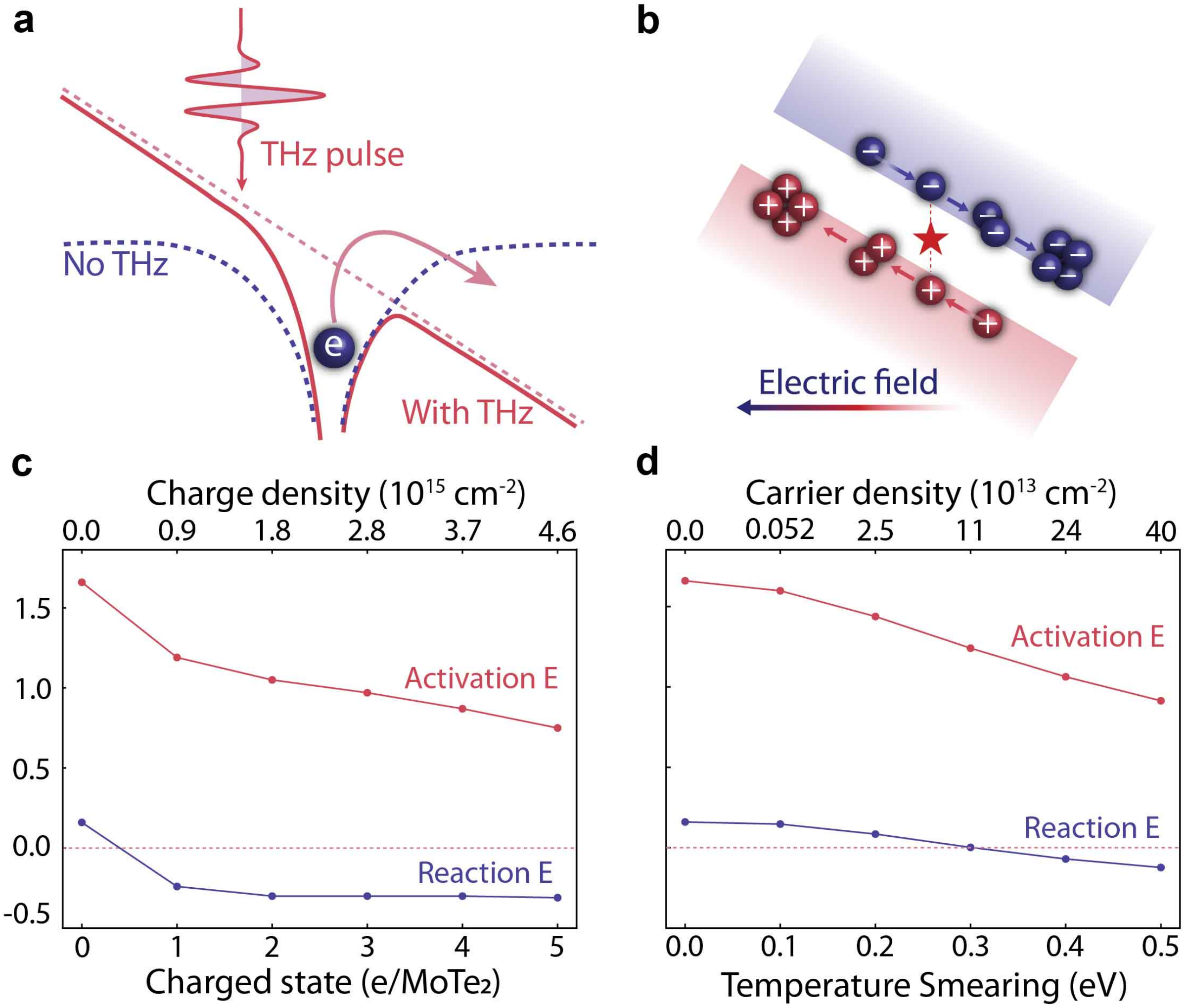

Supplementary Figure 17. **THz-driven carrier excitation and DFT-calculated energy landscapes.** **a**, Poole-Frenkel carrier liberation process in the presence of a THz electric field. Trapped carriers can escape to the conduction band because the potential barrier changes with a THz field. **b**, Impact ionization by collisions between THz-accelerated carriers and valence electrons, which creates new electron-hole pairs. These secondary carriers can also be accelerated to high energy, induce further impact ionization, and trigger an avalanche effect that increases the carrier density heavily. **c**, The activation energy (energy difference between $2H/2H^*$ and transition state) and reaction energy (energy difference between $2H/2H^*$ and $1T'$) at different charged states. For each charged state, the activation energy is obtained from the lowest barrier path. **d**, The activation energy and reaction energies at different temperature smearings.

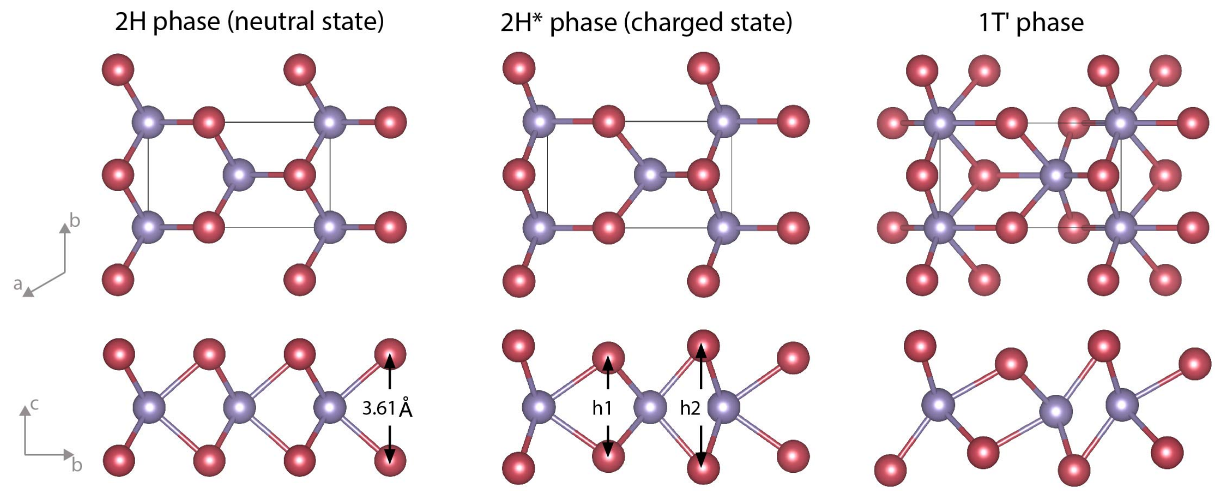

Supplementary Figure 18. **Atomic structures of monolayer $MoTe_2$ in the $2H$, $2H^*$, and $1T'$ phases.** The $2H$ phase undergoes a barrierless rearrangement to the $2H^*$ phase in charged states. In the $2H^*$ structure, h1 (h2) decreases (increases) almost linearly from 3.61 Å to 3.54 (3.69) Å to 3.26 (4.08) Å when the charge density increases from zero doping to 4.5×10^{14} cm^{-2} (0.5 e/MoTe$_2$), to 4.5×10^{15} cm^{-2} (5.0 e/MoTe$_2$). The $2H^*$ phase is not observed in the temperature smearing simulation.

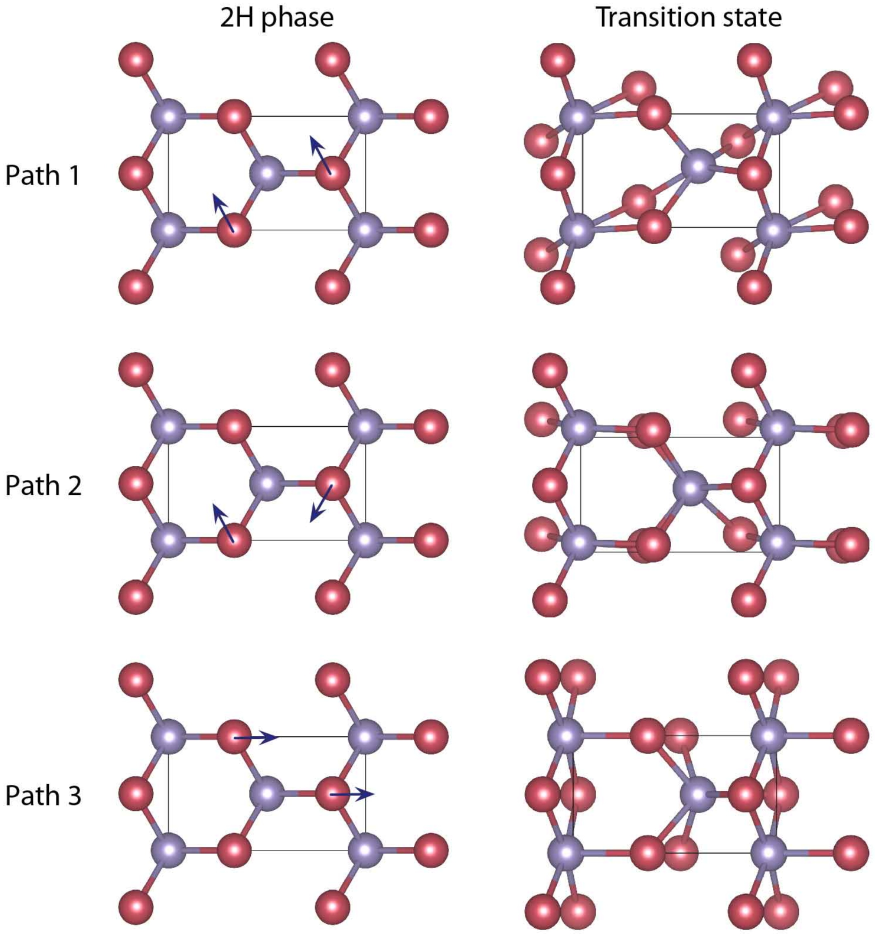

Supplementary Figure 19. **Considered paths of phase transition.** We calculated three possible paths. Blue arrows in the $2H$ phase structures indicate the largest components of atomic motion in the phase transition. The displayed transition state structures are from neutral state calculations.

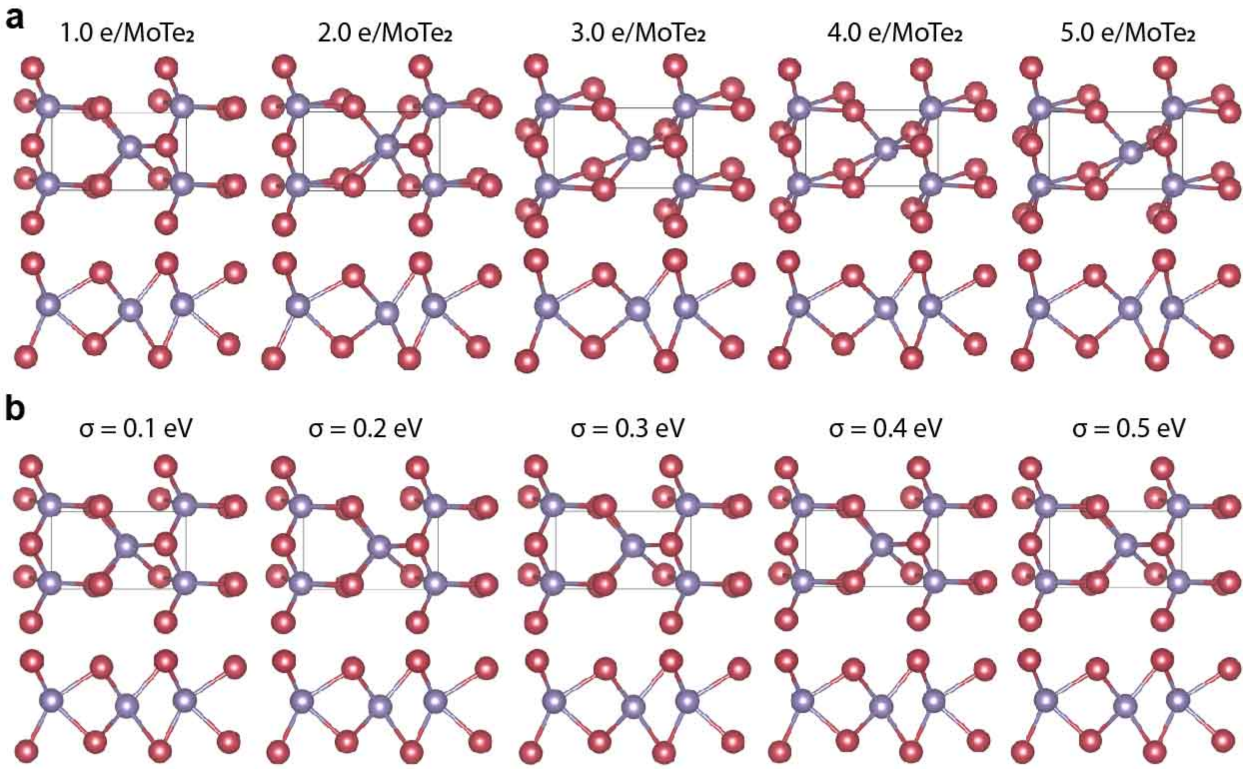

Supplementary Figure 20. **Dependence of transition state structures on the added charge density and Fermi-smearing width.** Among the three paths calculated, we chose the lowest barrier path to show its transition state structure. **a**, The neutral state, 9×10^{14} cm^{-2} (1.0 e/MoTe$_2$), and 1.8×10^{15} cm^{-2} (2.0 e/MoTe$_2$) cases prefer Path 2, and the remaining prefer Path 1 (see paths in Supplementary Fig. 19). **b**, All the cases with different Fermi-smearing widths prefer Path 2.

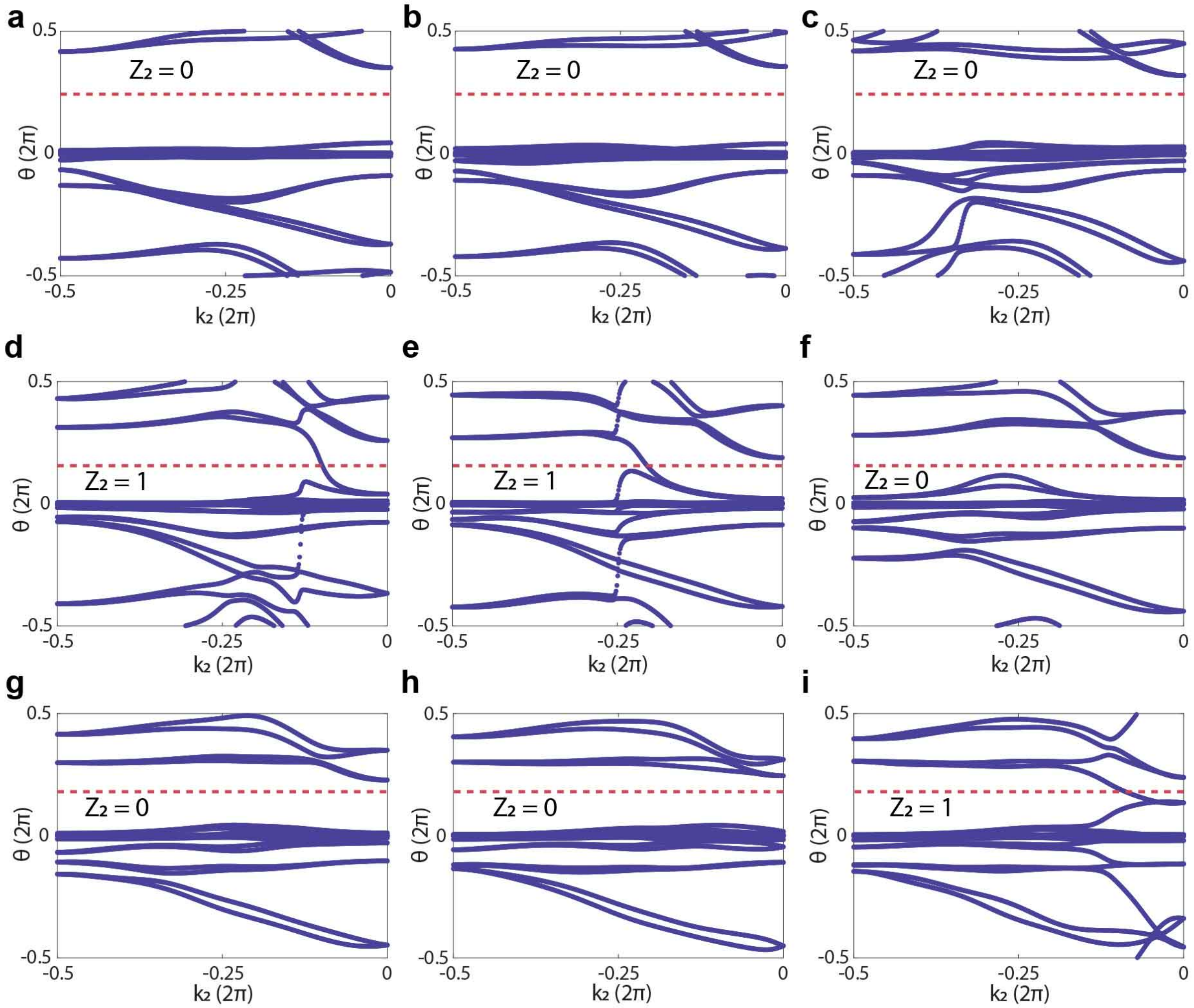

Supplementary Figure 21. **Wilson loop analysis of Z_2 topological index for $MoTe_2$.** Calculations were done for the $2H$ structure (**a**), the intermediate states (**b**–**h**), and the $1T'$ structure (**i**). The topologically non-trivial states ($Z_2 = 1$) can be distinguished from topologically trivial states ($Z_2 = 0$) by the odd or even number of crossings at the horizontal dashed red line.

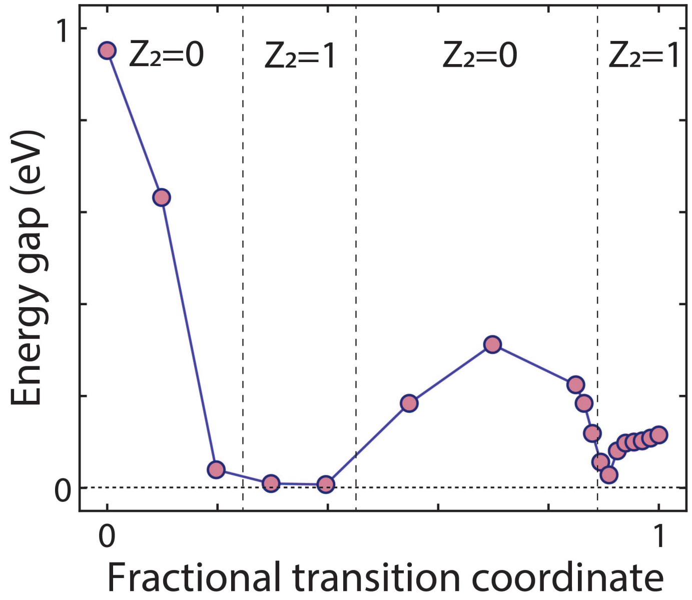

Supplementary Figure 22. **Evolution of the energy gap and Z_2 topological index along the $2H$-$1T'$ phase transition pathway.**

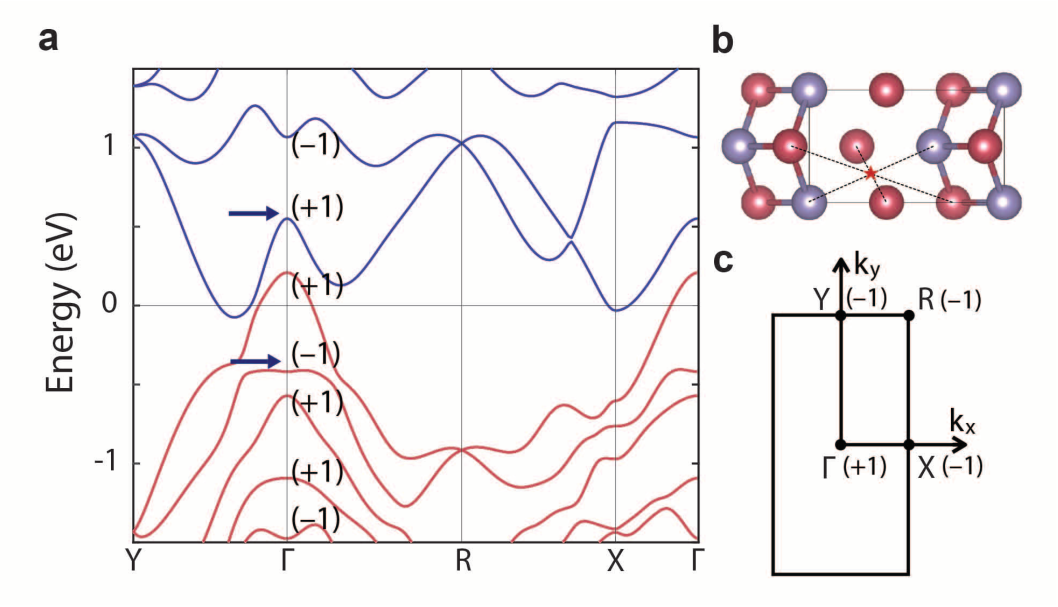

Supplementary Figure 23. **Analysis of Z_2 topological index.** **a**, Fu-Kane parity analysis of Z_2 topological index. The electronic band structure for the $1T'$ monolayer $MoTe_2$. The inversion symmetry eigenvalues for bands near the Fermi level at Γ point are labeled. The topological index is computed with the filled bands in red. **b**, Top view for the monolayer crystal with the inversion symmetry center (red star). **c**, The product for the inversion symmetry eigenvalues of the filled bands at the four time reversal invariant momentum (TRIM) points. The (−1) product indicates a non-trivial Z_2 topological phase for $1T'$ phase.

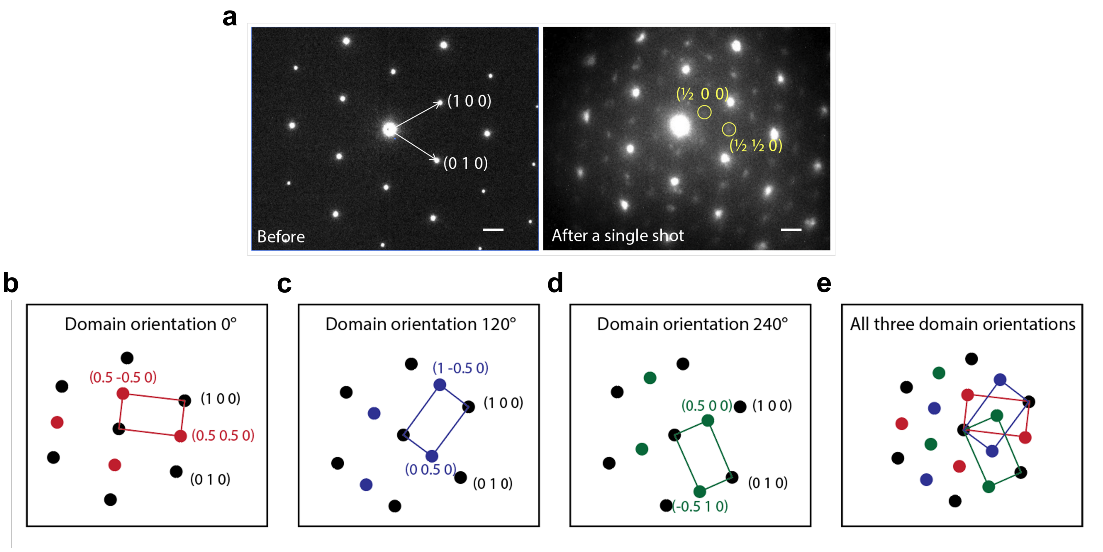

Supplementary Figure 24. **A comparison of our diffraction results with that in literature.** **a**, Electron diffraction pattern before and after a single THz shot with the electron beam direction incident along the [0 0 1] zone axis. The scale bars added to the plot represent 0.1 Å^{-1}. **b-e**, The first, second, and third calculated diffraction pattern comes from the $1T'$ phase with each domain rotated by a step of 120°, as described by ref.[4]. The emerged new peaks are labeled based on the original $2H$-$MoTe_2$ reciprocal lattice vector. Since the THz-induced $1T'$-$MoTe_2$ is multi-crystalline with three domains that are azimuthally rotated by 120°, their superposition yields the observed diffraction pattern as shown in **a** after the single shot.
